# Diclofenac Removal by Alkylammonium Clay Minerals Prepared over Microwave Heating

**DOI:** 10.1021/acsomega.4c05763

**Published:** 2024-11-23

**Authors:** Denise B. França, Alice P. N. Silva, Josy A. Osajima, Edson C. Silva-Filho, Santiago Medina-Carrasco, Maria del Mar Orta, Maguy Jaber, Maria G. Fonseca

**Affiliations:** † Universidade Federal da Paraíba, Núcleo de Pesquisa e Extensão - Laboratório de Combustíveis e Materiais (NPE - LACOM), Cidade Universitária s/n − Campus I, 58051-900 João Pessoa, PB, Brazil; ‡ Universidade Federal do Piauí, Laboratório Interdisciplinar de Materiais Avançados (LIMAV), Avenida Universitária s/n, 64049-550 Teresina, PI, Brazil; § Universidad de Sevilla, SGI Laboratorio de Rayos X - Centro de Investigación, Tecnología e Innovación de la Universidad de Sevilla (CITIUS), Avenida Reina Mercedes, 4B, 41012 Sevilla, Spain; ∥ Universidad de Sevilla, Departamento de Química Analítica da Facultad de Farmacia, Calle Profesor García González 2, 41012 Sevilla, Spain; ⊥ Sorbonne Université, CNRS UMR 8220, Laboratoire d’Archéologie Moléculaire et Structurale (LAMS), Case courrier 225, 4 pl. Jussieu, 75252 Paris Cedex 05, France

## Abstract

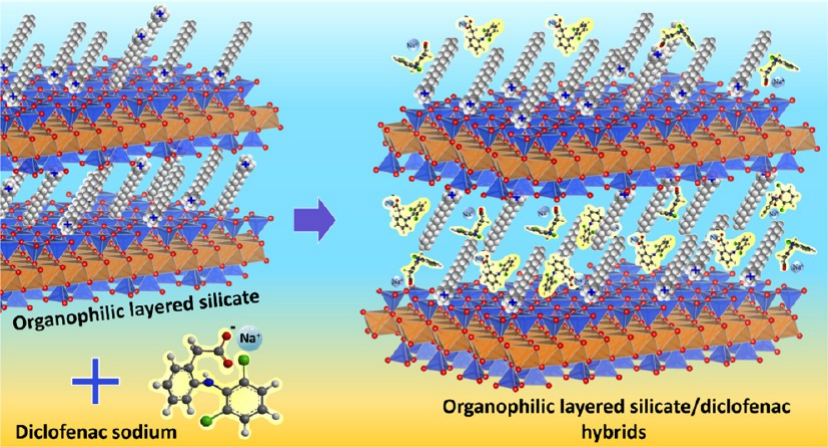

Diclofenac is an emerging contaminant widely detected in water and has had adverse effects on the biota. In this study, the adsorbents were prepared by reacting tetradecyl-(C_14_), hexadecyl-(C_16_), and octadecyltrimethylammonium (C_18_) bromides with sodium vermiculite (Na-Ver) and used for the removal of the first time for diclofenac sodium from aqueous solution. Synthesis was carried out in a microwave-assisted reactor operating at 50 °C for 5 min, using proportions of organic salts in 100 and 200% of the phyllosilicate cation exchange capacity. The stability of loaded alkylammonium solids was evaluated under drug adsorption conditions. Adsorption was mainly influenced by the amount of surfactant incorporated into the clay mineral according to the thermogravimetric and CHN elemental analysis data. Samples prepared with 200% CEC presented lower stability at pH 6.0 and 8.0. Drug adsorption was more effective for C_14_-Ver-200%, C_16_-Ver-200%, and C_18_-Ver-200% samples, with a maximum retention of 97.8, 110.1, and 108.0 mg g^–1^, respectively. The adsorptive capacities of C_14_-Ver-200%, C_16_-Ver-200%, C_18_-Ver-200%, C_14_-Ver-100%, C_16_-Ver-100%, and C_18_-Ver-100% were reduced to 29.0, 36.8, 41.0, 61.0, 50.4, and 58.0%, respectively, compared with their initial value after three adsorption cycles. X-ray diffraction (XRD) patterns revealed that diclofenac was adsorbed into the interlayer region of organovermiculites. Fourier transform infrared spectroscopy (FTIR), Zeta potential results, and the pH study of adsorption indicated that van der Waals interactions are dominant in the adsorption mechanism.

## Introduction

Nonsteroidal anti-inflammatory drugs (NSAIDs) are among the groups of pharmaceuticals that threaten the ecosystem and human health due to their presence in water.^1,2^ Sodium diclofenac (sodium 2-[2-(2-dichloroanilino) phenyl]acetate) is an NSAID highly consumed by hundreds of tons annually around the world for both human and veterinary medical care.^3,4^ The drug is among the most frequently detected in aquatic environments and has been involved in the European Union’s top 10 priority list for detection.^1,3,4^ The average concentrations of the drug in aquatic environments were higher than 0.1 μg L^–1^ in surface waters in Europe,^5^ while the concentration in Brazilian waters was 759.06 μg L^–1^.^6^

Diclofenac can lead to adverse effects on aquatic organisms,^7,8^ and the byproducts formed through biotic and abiotic transformations can pose even greater toxicity than the original molecule.^9,10^ Therefore, it is imperative to remove diclofenac from aquatic ecosystems. Adsorption is an interesting water treatment method due to its simplicity, cost-effectiveness, and high removal efficiency of pollutants, in addition to the absence of byproduct generation.^1^

Clay minerals are versatile, cheap, and highly available materials that can be used as adsorbents for drugs, among which montmorillonite (Mt) has been widely used for this proposal.^11,12^ More recently described in the literature, vermiculite is a clay mineral that also acts as an adsorbent for drugs.^13−19^ Vermiculite is a 2:1 phyllosilicate that exhibits an idealized negative layer charge per formula unit (ca. 0.6–0.9) and exchange cations in the interlayer region, normally Mg^2+^.^20^ The clay mineral is characterized by having tetrahedral silicate sheets that can be substituted by aluminum or other elements of lower valency, while the octahedral sites are generally occupied by Al^3+^, Mg^2+^, and Fe^3+^.^20^ Furthermore, vermiculite is a two-dimensional (2D) material^21^ that can be modified through the intercalation of organic compounds,^13,14^ acid activation,^22^ silylation,^23^ among others, to obtain materials with desired properties.

The adsorption of anionic drugs is significantly restricted in untreated clay mineral.^15,16^ However, drug adsorption performance of clay minerals can be further significantly improved by reacting with surfactants.^11,13−15^ Organovermiculites prepared with 1,3-2(hexadecamide propyl dimethylammonium chloride) *n*-butane, 1,3-2(hexadecamide propyl dimethylammonium chloride)-2-hydroxypropane dichloride, and 1,3-2(hexadecamide propyl dimethylammonium chloride)-*p*-xylene exhibited ibuprofen adsorption capacities of 322.6, 404.7, and 489.9 mg g^–1^, respectively, while drug adsorption by sodium vermiculite was negligible.^14^

Organoclays based on bentonite^24−27^ or montmorillonite,^28−31^ kaolinite,^27,32^ halloysite,^33^ Illite,^31^ and sepiolite^34^ were investigated as adsorbents for diclofenac sodium. In addition to clay minerals, other materials such as hydroxyapatite@chitosan hybrids,^35^ carbons,^36,37^ zeolites,^38^ carbon sphere@polyaniline@layered double hydroxides composites,^39^ and metal–organic frameworks^40,41^ were also studied.

For organoclays performance, the effects of experimental parameters such as pH, adsorbent dosage, time, temperature, drug concentration, and ionic strength on the diclofenac adsorption were evaluated.^24,26,28,42^ Although the drug has been detected in the environment at concentrations in the μg L^–1^ range, studies have been carried out at higher concentrations (10–2000 mg L^–1^) to understand the mechanisms of adsorption and factors that alter the performance of adsorbents.^27,28,32^ The effect of the type and amount of surfactant loading in the clay mineral matrix on the diclofenac adsorption performance was also evaluated.^24,28−31^ In summary, the increase in surfactant loading in the clay mineral improved the diclofenac adsorption.^24,25,29−31,43^

Despite extensive research in organoclays, the stability of the matrixes under drug adsorption conditions has been neglected and very few studies investigated the regeneration of these adsorbents.^25,33^ Since surfactant leaching is one of the factors that control organoclay-induced ecotoxicity,^44^ its stability must be known. The influence of pH on the stability of organovermiculites prepared with hexadecyltrimethylammonium and hexadecylpyridinium at 100% CEC for the adsorption of naphthalene was reported.^45,46^ Results showed that both samples were stable in the pH range of 4–10.^45^ However, the effect of the chain size and the amount of loaded surfactant on the stability of the organophilic clay mineral was not evaluated.

In the current investigation, a Brazilian vermiculite sample was modified with alkyl trimethylammonium salts with different chain lengths (C_14_, C_16_, and C_18_) by microwave heating (MW) and used for the first time as adsorbents for sodium diclofenac, a predominantly anionic drug at pH 6.0 (p*K*_a_ = 4.15). Reactions over MW heating have been shown to be an effective route for the modification of clay minerals.^47,48^ Brazil has abundant reserves of vermiculite,^49^ as well as a demand for sodium diclofenac, which has been detected in Brazilian surface waters,^6,50,51^ due to the inefficiency of conventional water treatment methods used.^6^ No studies regarding the use of organophilic vermiculites for the adsorption of diclofenac have been verified until this point. The evaluation of diclofenac adsorption by the resulting materials was carried out under varying experimental conditions that included pH levels, adsorbent dosage, contact time, and diclofenac concentrations. The stability of organophilic vermiculites under adsorption conditions was also verified. Therefore, the impacts of the composition and size of the alkyl chain of surfactants on the stability of organovermiculites and diclofenac adsorption performance of diclofenac were evaluated, and the potential for reusing organoclay was also explored.

## Results and Discussion

### Characterizations

#### X-ray Diffractometry

**Figure 1:**
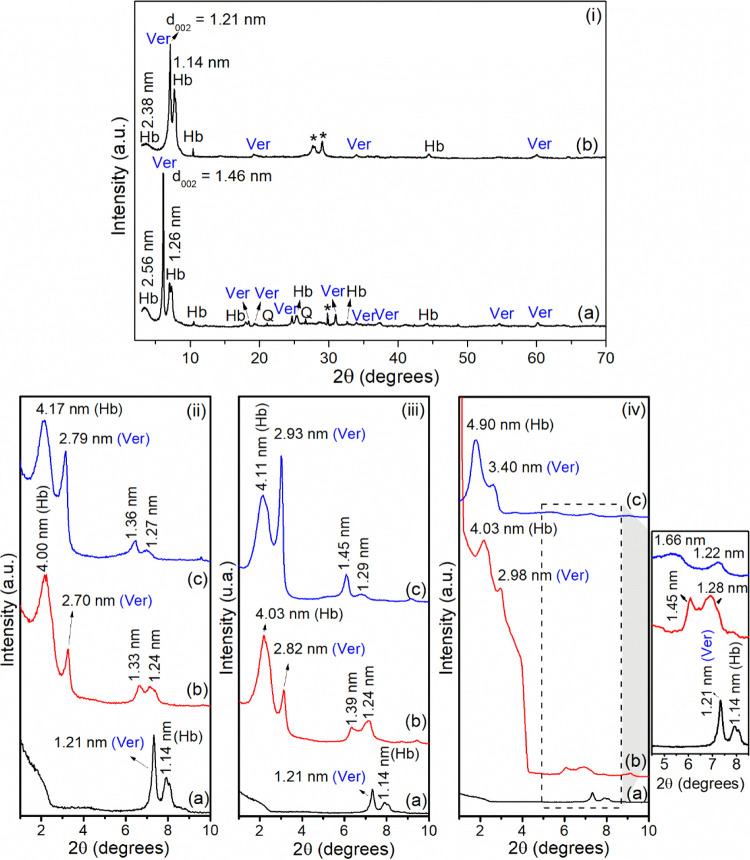
XRD patterns of (i): (a) Ca,Mg-Ver and (b) Na-Ver (Ver = vermiculite, Hb = hydrobiotite, Q = quartz, *unidentified phase); (ii): (a) Na-Ver, (b) C_14_-Ver-100%, and (c) C_14_-Ver-200%; (iii): (a) Na-Ver, (b) C_16_-Ver-100%, and (c) C_16_-Ver-200%; and (iv): (a) Na-Ver, (b) C_18_-Ver-100%, and (c) C_18_-Ver-200%.

The X-ray diffractometry (XRD) patterns of Ca,Mg-Ver, Na-Ver, and organovermiculites are presented in Figure 1. The results suggested that the Ca,Mg-Ver sample (Figure 1i-a) is composed of vermiculite (ICDD 00-034-0166), with impurities of hydrobiotite (ICDD 00-049-1057) and quartz (ICDD 00-046-1045). Hydrobiotite (Hb) is a regular interstratified biotite/vermiculite phase in a 1:1 proportion as a result of the weathering of micas,^20^ and its presence is frequently reported in vermiculite samples.^52,53^ The principal reflection of vermiculite occurred at 2θ = 6.13° (002 plane) and resulted in a basal spacing of 1.46 nm.^54,55^ The reflection at 2θ = 60.13° (*d* = 0.154 nm, 060 plane) was assigned to the trioctahedral clay mineral.^56^ For the Hb phase, reflections occurred at 2θ = 3.45° (*d* ∼ 2.56 nm, 001 plane) and 7.00° (*d* = 1.26 nm, 002 plane).^57^

After the Na^+^ exchange reaction, basal space changed to 1.21 nm (Figure 1i-b), as a result of the substitution of the interlayer cations in the raw sample (normally Mg^2+^) and the reduction of the water molecules in a monolayer arrangement in the interlayer region.^58^ In the Hb phase, the changes in *d*_001_ and *d*_002_, measuring 2.38 and 1.14 nm, respectively, align with the saturation of the samples with sodium following the exchange process.^59^

In organophilic samples, two reflections were observed at 2θ < 4.0° (Figures 1ii–iv) corresponding to basal distances ranging between 2.70–3.40 nm and exceeding 4.00 nm which could be due to intercalation of alkylammonium cations in vermiculite and hydrobiotite, respectively.^60,61^ The basal distances increased with the chain size of surfactants C_14_ (2.27 nm), C_16_ (2.53 nm), and C_18_ (2.79 nm).^62^ Considering that the 2:1 layer thickness is about 0.96 nm^20^ and based on the basal spacings and the surfactant size, the intercalation of organic cations in paraffin-like monolayer arrangements is proposed for all organophilic samples, for both Ver and Hb phases.^60^ An illustration of this conformation is presented in Figure S1. For the Ver phase, second-order reflections were also observed at 1.33–1.36, 1.39–1.45, and 1.45–1.66 nm for C_14_-Ver, C_16_-Ver, and C_18_-Ver, respectively.^63^

#### Thermogravimetry (TG/DTG)

**Figure 2:**
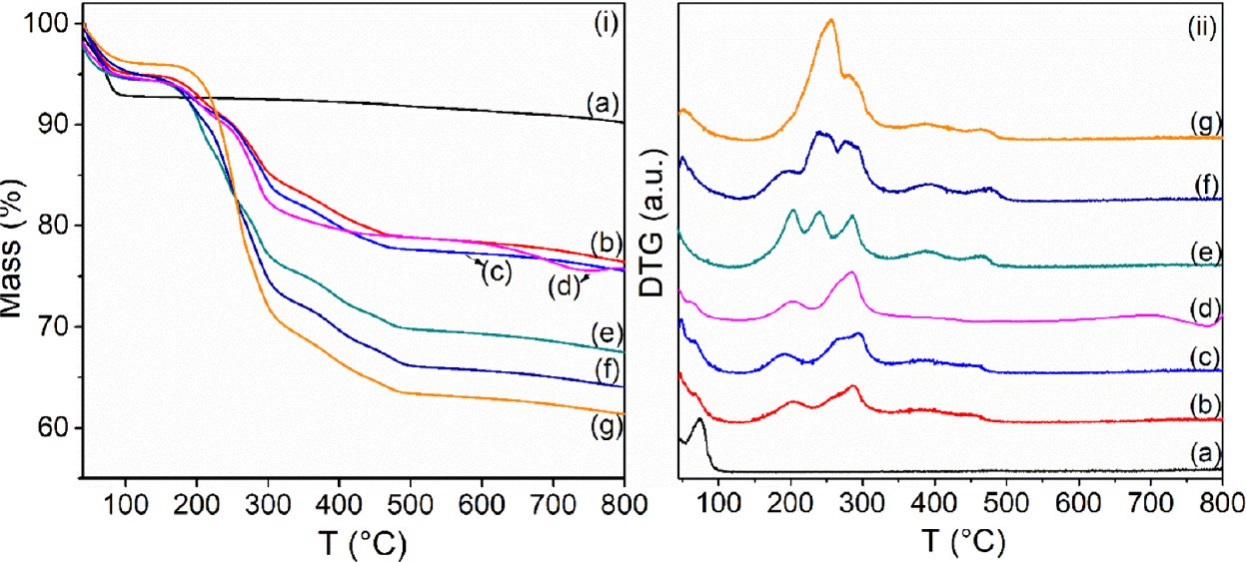
(i) TG and (ii) DTG curves for (a) Na-Ver, (b) C_14_-Ver-100%, (c) C_16_-Ver-100%, (d) C_18_-Ver-100%, (e) C_14_-Ver-200%, (f) C_16_-Ver-200%, and (g) C_18_-Ver-200%.

TG/DTG was used for the quantification of the organic content in the organophilic vermiculites. Results are shown in Figure 2 and summarized in Table 1. For the Na-Ver sample, the curve exhibited two thermal decomposition events, resulting in 9.4% total mass losses in the 30–800 °C range, while the organophilic samples presented 23.6 to 38.6% total mass losses in the same temperature range. The initial mass loss event for Na-Ver (30–125 °C) corresponded to the loss of the physically adsorbed Na-Ver on the clay mineral surface. The subsequent mass loss (366–800 °C) is associated with the condensation of silanol groups of the Ver and Hb phases.^64,65^

**Table 1:** Summary of Mass Losses and Temperature Intervals Based on DTG Curves for Na^+^-Ver and Organovermiculites

sample	event	*T* (°C)	mass loss (%)	total mass loss (%)	total organic content^a^ (%)
Na-Ver	I	30–125	7.2	9.4	
	II	366–800	2.2		
C_14_-Ver-100%	I	30–126	5.0	23.6	16.2
	II	126–232	3.9		
	III	235–335	7.4		
	IV	335–433	3.7		
	V	433–522	1.3		
	VI	522–800	2.3		
C_14_-Ver-200%	I	30–120	5.5	32.5	24.8
	II	120–221	6.9		
	III	221–262	5.6		
	IV	262–337	6.5		
	V	337–436	4.0		
	VI	436–532	1.8		
	VII	532–800	2.1		
C_16_-Ver-100%	I	30–130	5.6	24.5	16.8
	II	126–223	3.2		
	III	223–340	8.9		
	IV	340–435	3.5		
	V	435–504	1.1		
	V	570–800	2.1		
C_16_-Ver-200%	I	30–128	5.2	36.0	28.8
	II	128–210	4.6		
	III	210–267	9.8		
	IV	267–343	8.2		
	V	343–440	4.1		
	VI	440–519	2.1		
	VII	573–800	2.0		
C_18_-Ver-100%	I	30–126	4.4	24.5	17.0
	II	126–229	4.5		
	III	229–363	10.4		
	IV	363–459	1.4		
	V	459–517	0.7		
	VI	517–779	3.0		
C_18_-Ver-200%	I	30–138	4.0	38.6	32.8
	II	138–275	19.4		
	III	275–348	7.6		
	IV	348–442	4.1		
	V	442–537	1.6		
	VI	507–800	1.9		

a Values were obtained considering the sum of mass losses in the events, excluding dehydration and dehydroxylation.

For organophilic samples, the decrease in mass loss during the initial event suggests an enhancement in hydrophobicity.^63,66^ The mass loss events in the range of about 120–537 °C were assigned to the decomposition of organic cations incorporated in the clay mineral and were used to estimate the percentage of organic content in the organophilic samples (see Table 1). Higher percentages of organic content (24.8–32.8%) were observed for organovermiculites prepared with surfactant amounts of 200% CEC. The final thermal decomposition event was related to structural hydroxyl condensation, and organophilic samples did not show significant differences compared to Na-Ver.^63,67^

#### CHN Elemental Analysis

The quantification of surfactants incorporated in organophilic samples was also performed by using CHN elemental analysis (Table 2). The total percentage of the values of organic content was close to those obtained by the TG/DTG analysis. The amounts of surfactants in the C_14_-Ver-100%, C_16_-Ver-100%, and C_18_-Ver-100% samples were close to the initial values used in their preparation (0.67 mmol/g), and high organic incorporations were obtained for the surfactant proportions at 200% CEC.

**Table 2:** Results of CHN Elemental Analysis of Organophilic Vermiculites

	C		H	N		α^a^	Q^b^
sample	(%)	(mmol/g)	(%)	(%)	(mmol/g)	(%)	(mmol/g)
C_14_-Ver-100%	11.6	9.7	3.2	0.9	0.6	15.7	0.6
C_16_-Ver-100%	13.0	10.8	3.3	0.9	0.7	17.2	0.7
C_18_-Ver-100%	14.1	11.8	3.6	0.8	0.6	18.5	0.6
C_14_-Ver-200%	18.2	15.2	4.3	1.4	1.0	23.9	1.0
C_16_-Ver-200%	21.3	17.8	4.8	1.4	1.0	27.5	1.0
C_18_-Ver-200%	23.8	19.8	5.2	1.4	1.0	30.3	1.0

a Total organic content determined from CHN elemental analysis.

b Amount of surfactant in the samples.

#### FTIR Spectroscopy

**Figure 3:**
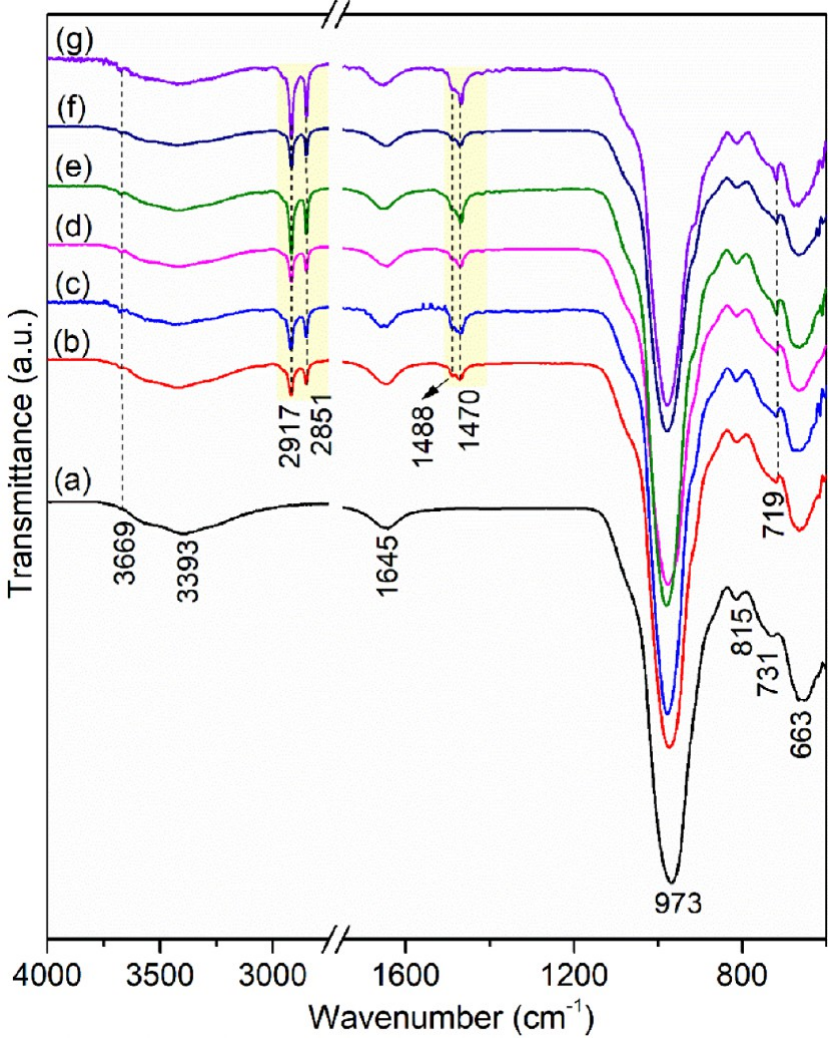
FTIR spectra of (a) Na-Ver, (b) C_14_-Ver-100%, (c) C_14_-Ver-200%, (d) C_16_-Ver-100%, (e) C_16_-Ver-200%, (f) C_18_-Ver-100%, and (g) C_18_-Ver-200%.

Infrared spectroscopy is widely used to obtain qualitative information about the organophilization of clay minerals with surfactants, as well as the conformation of ammonium cations in the interlayer region.^68^ The FT-IR spectra of Na-Ver and organophilic vermiculites are shown in Figure 3. The spectrum of the Na-Ver sample (Figure 3a) shows a shoulder at 3669 cm^–1^, attributed to the OH stretching of the structural groups of clay minerals, and a broad band at 3393 cm^–1^, assigned to the OH stretching vibrations of water molecules.^54^ The band at 1645 cm^–1^ is associated with the deformation vibrations of water molecules.^57^ Bands at 973 and 815 cm^–1^ were assigned to Si–O stretchings, while bands at 731 and 683 cm^–1^ are linked to the in-plane deformation vibration of Al–O–Si bonds.^54^

The presence of new bands in the infrared spectra assigned to the surfactants was observed in all organophilic vermiculites (Figure 3b,g). Bands at 2917 and 2851 cm^–1^ were attributed to antisymmetric and symmetric stretchings in the CH_2_ groups, respectively.^14^ These bands are very close to the free surfactant frequency, observed at 2916 and 2849 cm^–1^, indicating that the organic chains adopt an ordered conformation (all-trans conformation) in organovermiculites.^68^ The band at 1488 cm^–1^ was related to CH_3_ deformation, while the bands in 1471–1469 and 719 cm^–1^ were assigned to CH_2_ deformation.^68^

#### Electron Microscopy

**Figure 4:**
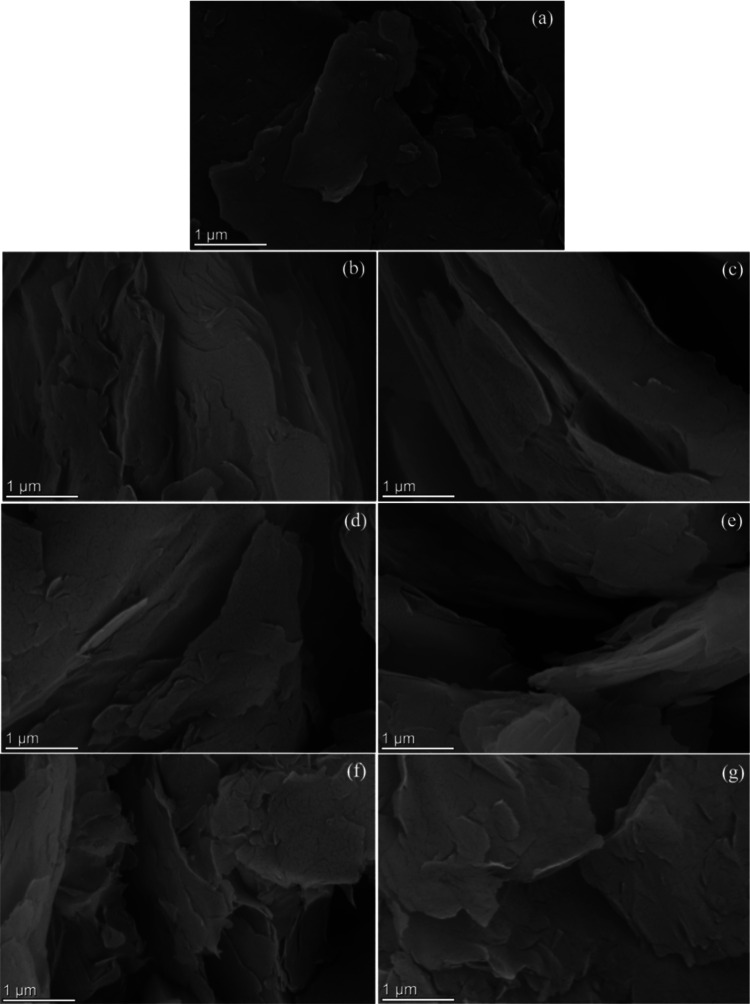
SEM images of (a) Na-Ver, (b) C_14_-Ver-100%, (c) C_14_-Ver-200%, (d) C_16_-Ver-100%, (e) C_16_-Ver-200%, (f) C_18_-Ver-100%, and (g) C_18_-Ver-200%.

Morphology of the Na-Ver and organophilic vermiculites was followed by scanning electron microscopy (SEM) and transmission electron microscopy (TEM) analysis. SEM images of the Na-Ver and organophilic vermiculites are shown in Figure 4. Na-Ver exhibited a plate-like morphology characteristic of the clay mineral,^69^ which was maintained after modification with surfactants.

**Figure 5:**
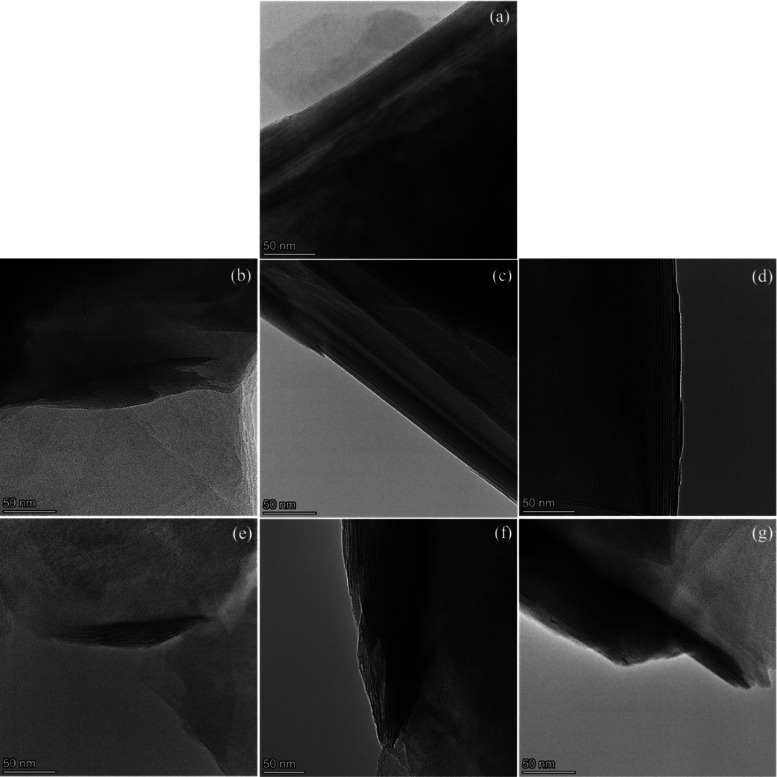
TEM images of (a) Na-Ver, (b) C_14_-Ver-100%, (c) C_14_-Ver-200%, (d) C_16_-Ver-100%, (e) C_16_-Ver-200%, (f) C_18_-Ver-100%, and (g) C_18_-Ver-200%.

TEM images are presented in Figures 5 and S2. The interplanar distances for Na-Ver were measured at 1.0 and 1.1 nm, lower than the XRD basal space, potentially attributed to sample dehydration under vacuum.^70^ In organophilic samples, the basal spaces were larger than those observed for Na-Ver, indicating the intercalation of surfactants (Table S1). The values were close to those obtained by XRD; nevertheless, the *d* values ≥4.00 nm of the Hb phase were only evident in the TEM images for the C_16_-Ver-100% and C_18_-Ver-100% samples. In certain regions, organophilic samples displayed basal distances ranging from 1.0 to 1.2 nm, closely resembling those of the Na-Ver and hydrobiotite phase. This suggests that not all interlayers—clay mineral are intercalated by organic cations.^71^

#### Textural Properties

The N_2_ adsorption–desorption isotherms and textural parameters (specific surface area, pore volume, and pore diameter) of Na-Ver and organovermiculites (C_14_ and C_16_) prepared at 100% CEC are shown in Figure S3a–c and Table 3. For C_18_-Ver-100%, Kr adsorption was performed Figure S3d and the N_2_ isotherm was not obtained possibly due to the nature of the sample. The samples presented a type H3 loop in the IUPAC classification with no plateau at high *P*/*P*_0_ and this type indicates that the adsorption branch resembles a Type II isotherm and that the lower limit of the desorption branch is normally located at the cavitation-induced *P*/*P*_0_.^72^ H3 loops are given by nonrigid aggregates of plate-like particles like clay minerals.^72^ The specific surface area of Na-Ver is within the range reported for Santa Luzia vermiculite samples reported in the literature (16 to 34 m^2^ g^–1^),^57,69^ whose values depend on the size of the particle.^73^ The presence of surfactant in the samples decreased the specific surface area and the volume of the pores of the clay mineral, while the diameter of the pores increased. This behavior has also been reported for other organophilic vermiculites prepared with C_14_,^16^ C_16_^16,74^ and other surfactants,^66,75,76^ and occurs due to blockage of the structural pores of the clay mineral by incorporation of organic cations.^16,66^

**Table 3:** Textural Parameters of Na-Ver and Organovermiculites Prepared with 100% CEC

sample	*S*_BET_ (m^2^ g^–1^)	pore volume (cm^3^ g^–1^)	pore diameter (nm)
Na-Ver	29	0.064	13
C_14_-Ver-100%	4	0.031	35
C_16_-Ver-100%	3	0.027	34
C_18_-Ver-100%	3		

### Adsorption

#### pH Effect

**Figure 6:**
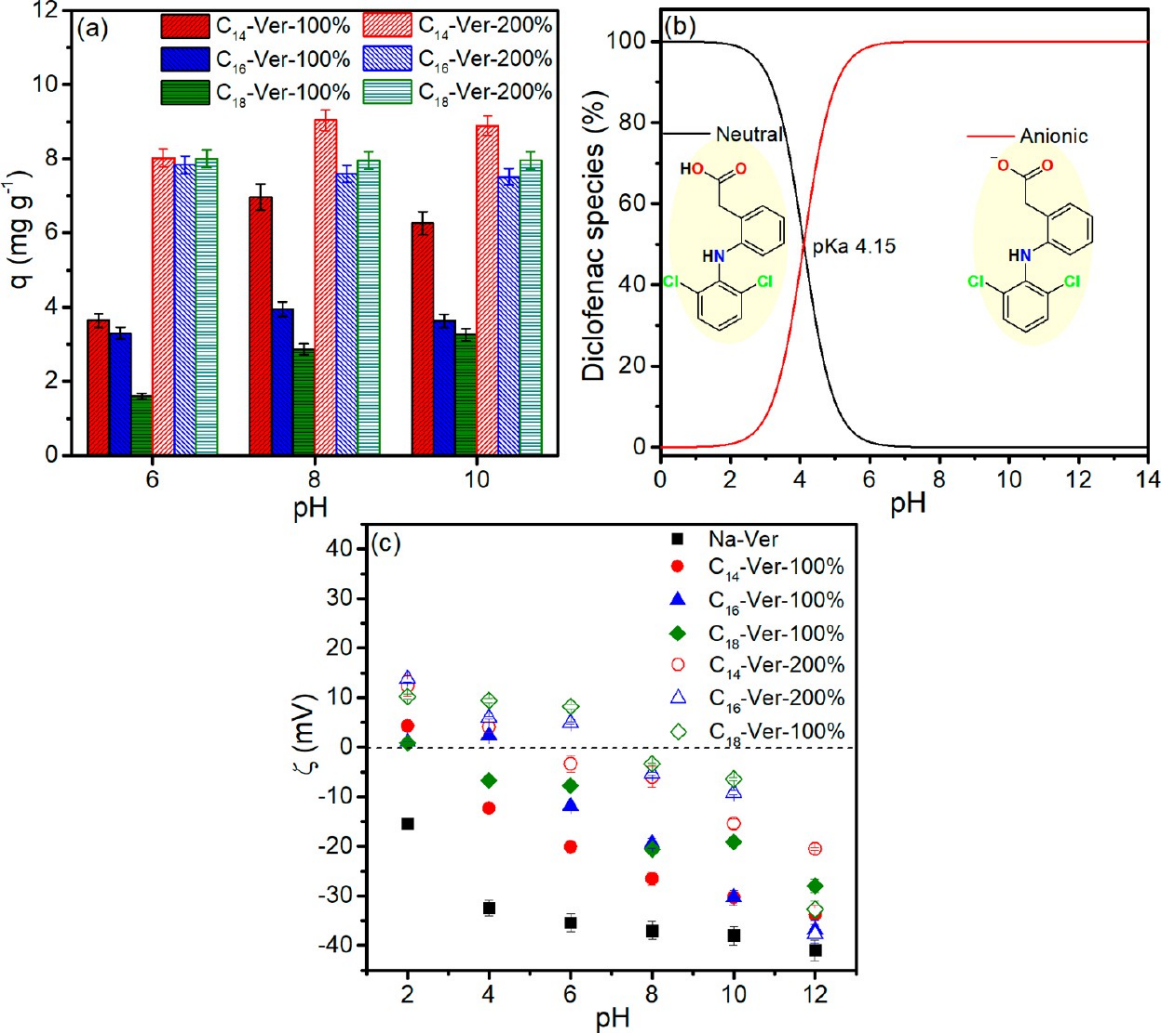
(a) Influence of pH on drug adsorption by organophilic vermiculites (conditions: 24 h, 25 °C, 25 mg mass adsorbent and *C*_i_ = 10 mg L^–1^), (b) diclofenac speciation as a function of pH and (c) zeta potential (ζ) measurements of Na-Ver and organophilic vermiculites.

The test results illustrating the influence of pH on sodium diclofenac adsorption by organophilic vermiculite are shown in Figure 6a. No adsorption experiments were carried out at pH < 6 to avoid precipitation of diclofenac due to the presence of the neutral form under this condition, as shown in the speciation diagram as a function of pH (Figure 6b), which is even less soluble than salt.^37^ The results demonstrate that adsorption was slightly higher at pH 8 for C_14_-Ver-100%, C_14_-Ver-200%, C_16_-Ver-100%, and C_18_-Ver-100%, producing values of 6.9, 9.0, 3.9, and 2.9 mg g^–1^, respectively. In the case of C_16_-Ver-200% and C_18_-Ver-200%, adsorption was independent of the pH range (6 to 10). Na-Ver did not exhibit diclofenac adsorption within the pH range; therefore, alkyl trimethylammonium salts provided sites for diclofenac adsorption on organophilic vermiculites.

The results of the Zeta potential measurements of Na-Ver and organophilic vermiculites are shown in Figure 6c. Na-Ver exhibits negative charge throughout the entire pH range due to isomorphic substitutions in the lattice, which generates a permanent negative charge on the surface,^77−79^ while the decrease in charge with increasing pH results from dissociation of the hydroxyl groups of the edge surfaces.^77^ On the other hand, diclofenac (p*K*_a_ 4.15) is predominately anionic at pH 6.0 (98.61%), 8.0 (99.98%), and 10.0 (99.99%), Figure 6b, which complicates adsorption on clay mineral due to repulsion between charges.

Organophilic vermiculites showed Zeta potential values higher than those of Na-Ver at the pH of adsorption, which reduces the repulsion by anionic species. The increase in surface charge occurs due to the adsorption of cationic surfactants on the negatively charged surface of the clay mineral.^80,81^ The results show that there was no relationship between the amount of diclofenac adsorbed and the surface charge of organophilic vermiculites (Figure S4), which suggests that van der Waals interactions between diclofenac and the alkyl chain of the surfactant played a role in the adsorption mechanism.^24^

#### Adsorbent Dosage

**Figure 7:**
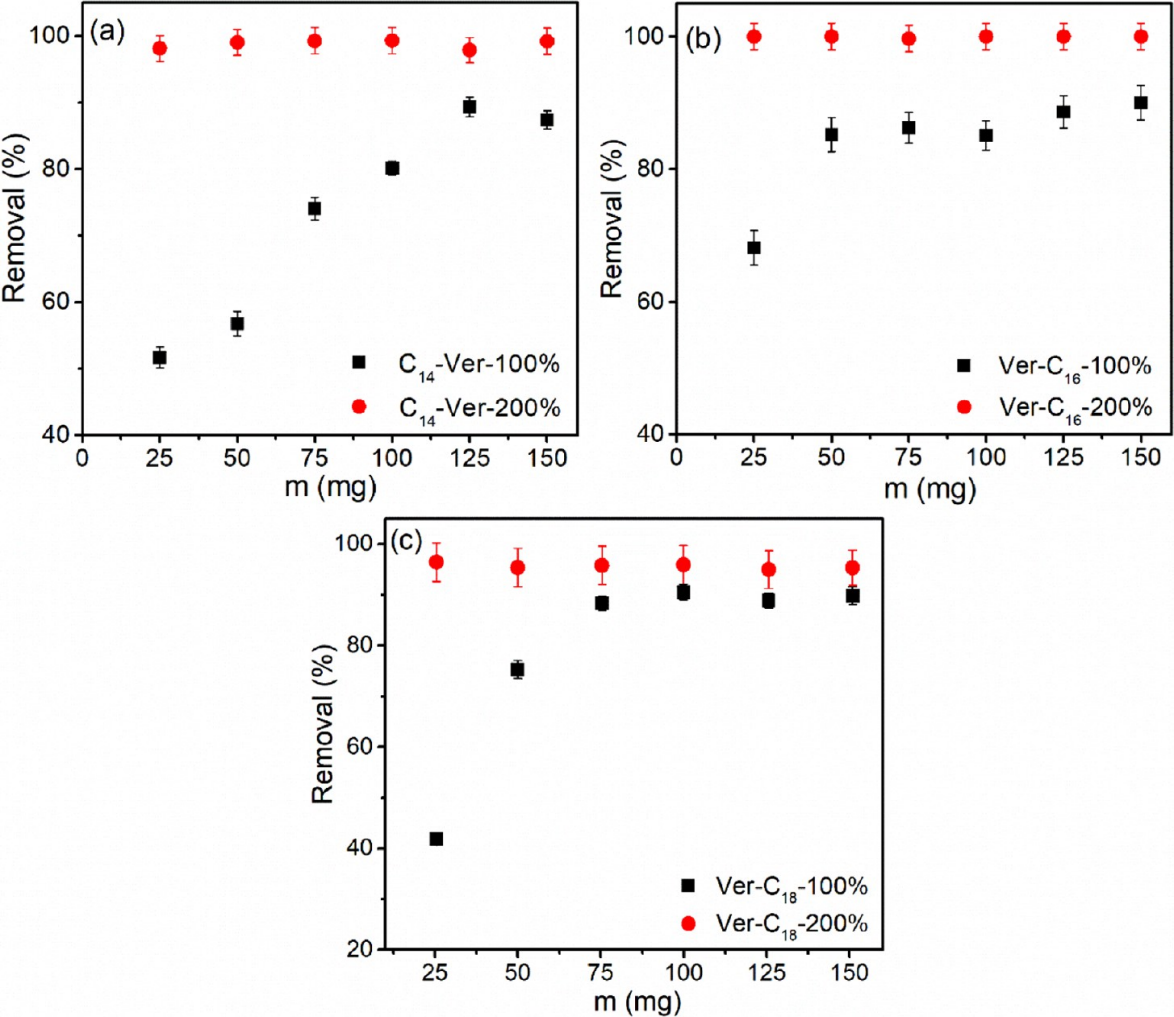
Effect of the dosage of the adsorbent on the adsorption of the drug by organovermiculites (a) C_14_-Ver, (b) C_16_-Ver, and (c) C_18_-Ver (24 h, pH 6.0 or 8.0, 25 °C and *C*_i_ = 10 mg L^–1^).

The influence of the dosage of the adsorbent on the removal of diclofenac by organophilic vermiculites (Figure 7) was studied under the optimal pH conditions obtained for each adsorbent. The findings revealed that the highest percentage of drug removal (96–99%) was observed for C_14_-Ver-200%, C_16_-Ver-200%, and C_18_-Ver-200% samples, achieved with 25 mg of each adsorbent. However, for C_14_-Ver-100%, C_16_-Ver-100%, and C_18_-Ver-100%, higher doses of 125, 50, and 75 mg were required, resulting in removal percentages of 89, 85, and 88%, respectively. This indicates that larger amounts of adsorbents were necessary for samples with lower surfactant contents, directly influencing the availability of adsorption sites.^82,83^

#### Adsorption Kinetics

**Figure 8:**
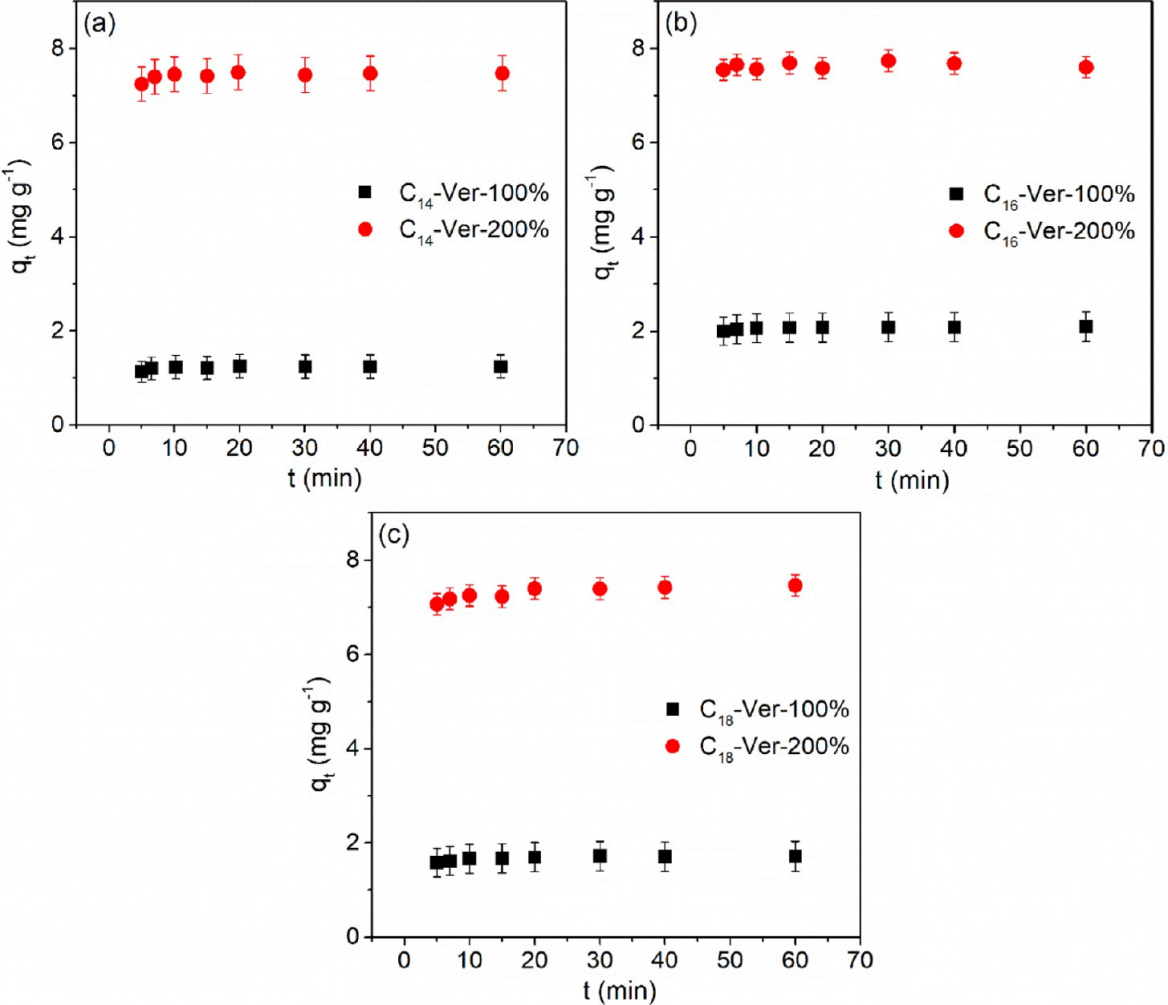
Kinetic adsorption isotherms for adsorption of diclofenac sodium by the organovermiculites (a) C_14_-Ver, (b) C_16_-Ver, and (c) C_18_-Ver (25 °C, pH 6.0 or 8,0 and *C*_i_ = 10 mg L^–1^).

The results obtained in the kinetic study (Figure 8) demonstrated that the drug was rapidly adsorbed on organophilic vermiculites at an equilibrium time of only 5 min for all hybrids. The result obtained was very close to that observed for diclofenac adsorption by modified C_16_Br kaolinites, ∼6 min,^43^ and was shorter than those obtained with other organophilic clay minerals, such as modified C_16_Br Mt (60 min),^25^ commercial organoclay Spectrogel Type C (500 min),^84^ and alkypyridinium bentonites (10 and 60 min).^24^

The fitting of experimental data to the adsorption kinetic models was not performed, because of rapid adsorption. The use of data at or very close to, equilibrium, is likely to lead to erroneous conclusions regarding adsorption kinetics.^85,86^

#### Adsorption Isotherms

**Figure 9:**
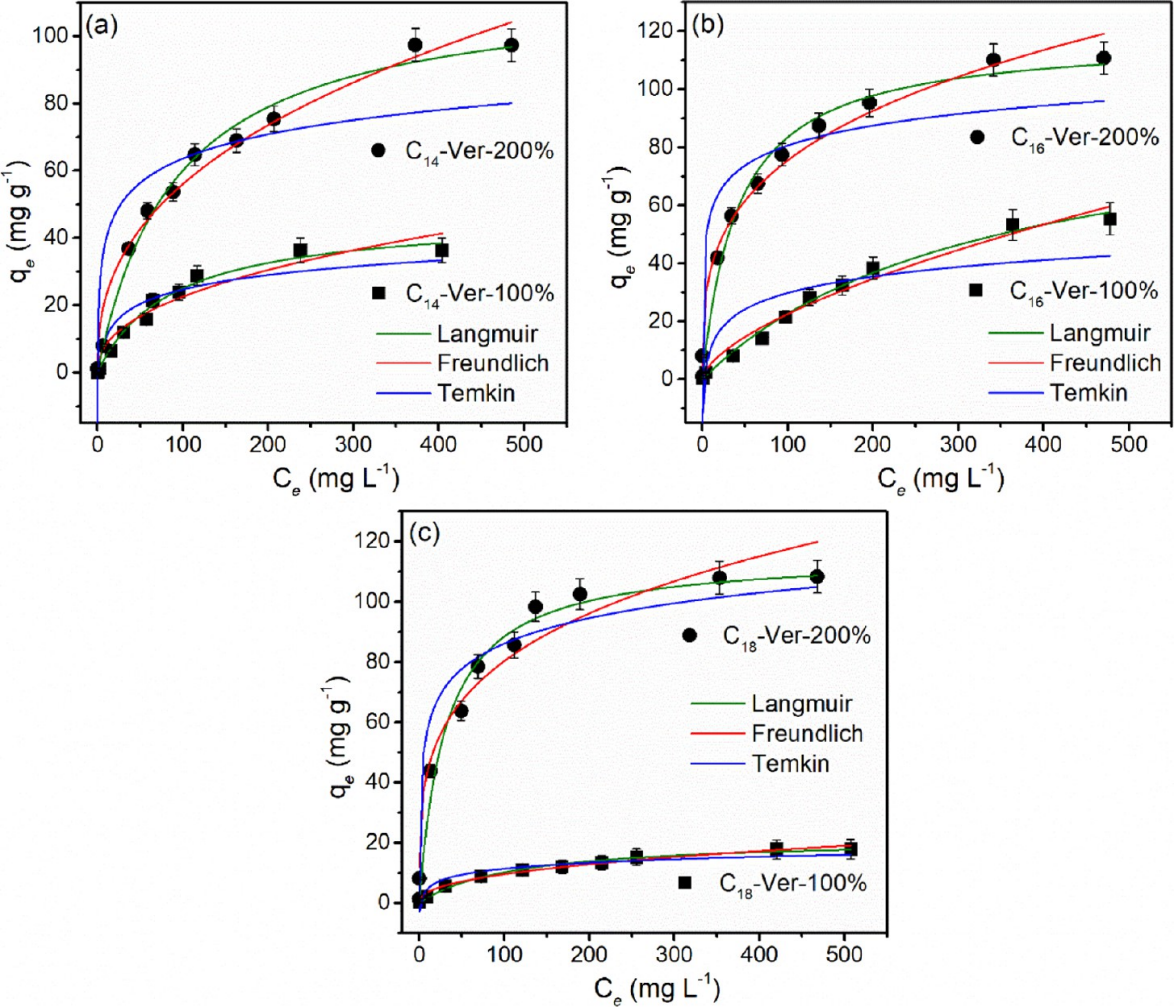
Equilibrium isotherms and their fit to the Langmuir, Freundlich, and Temkin models for the adsorption of diclofenac sodium by organovermiculites (a) C_14_-Ver, (b) C_16_-Ver, and (c) C_18_-Ver at 25 °C (pH 6.0 or 8.0, 25 °C and *C*_i_ = 1–500 mg L^–1^).

The adsorption isotherms are present in Figure 9 and the data were evaluated for adjustment to the Langmuir, Freundlich, and Temkin models; the parameters obtained are shown in Table S2. Taking into account *R*^2^ and SD, the experimental data were fitted to the Langmuir model for all investigated solids.

The adsorption isotherms illustrated a greater adsorption of diclofenac by organophilic vermiculites with an increasing initial concentration of the pollutant. The maximum adsorption capacities observed for the hybrids C_14_-Ver-200%, C_16_-Ver-200%, and C_18_-Ver-200% were 97.75, 110.6^,^ and 107.97 mg g^–1^, respectively, while the adsorption values were 36.30, 52.90, and 17.88 mg g^–1^ for C_14_-Ver-100%, C_16_-Ver-100%, and C_18_-Ver-100%.

Drug adsorption increased with the amount of surfactant in the C_14_-Ver, C_16_-Ver, and C_18_-Ver samples (Figure S5). These results are in agreement with data found for diclofenac adsorption in benzyldimethyltetradecylammonium-modified Mt,^29^ Mt and kaolinite modified by C_16_Br,^25,31,43^ and dodecy and hexadecylpyridinium-bentonites.^24^ Samples with a higher amount of surfactant incorporated have more available active sites for diclofenac interaction, improving the adsorption of pollutant.^27^

The number of carbons in the alkyl chain of the surfactants influenced the adsorption of the drug only for samples prepared with 100% CEC and the performance followed the order C_16_-Ver-100% > C_14_-Ver-100% > C_18_-Ver-100%. The difference in the adsorption of the C_14_-Ver-100%, C_16_-Ver-100%, and C_18_-Ver-100% samples can occur due to the contribution of a series of interrelated factors, such as the length of the alkyl chain, the packing density of the surfactants and the organic content of the samples in concordance with other organophilic clay minerals.^24,87^

The adsorption performance of the C_14_-Ver-200%, C_16_-Ver-200%, and C_18_-Ver-200% samples was better than that obtained by other organoclays prepared with higher amounts of surfactants (≥200% CEC),^24,28^ including pristine clay mineral with CEC close to sample used in the present study (Table S3). The CEC influences the amount of loaded surfactant into the clay mineral,^88,89^ and consequently the availability of drug adsorption sites.

#### Organovermiculites Stability

**Figure 10:**
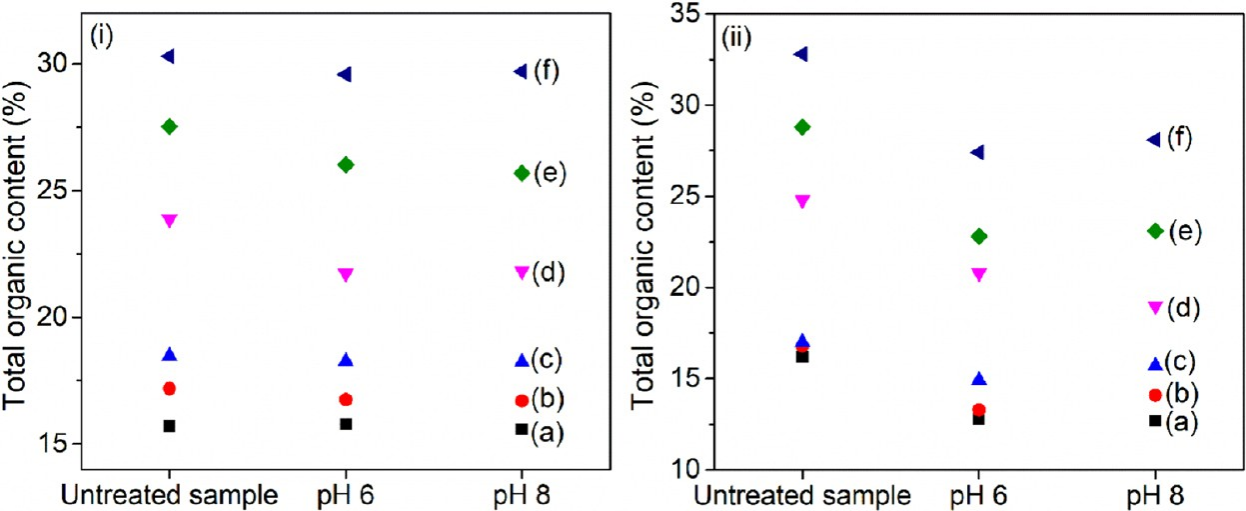
Total organic content of (a) C_14_-Ver-100%, (b) C_16_-Ver-100%, (c) C_18_-Ver-100%, (d) C_14_-Ver-200%, (e) C_16_-Ver-200%, and (f) C_18_-Ver-200% before and after stability test at pH 6.0 and 8.0 determined by (i) CHN and (ii) TG/DTG.

The stability of organophilic vermiculites after treatment at pH 6.0 and 8.0 was monitored by TG/DTG (Figure S6 and Table S4) and CHN elemental analysis (Table S5). Results indicated a small reduction in the total organic content of the samples after the stability test (Figure 10). The lower leaching of organic cations and the highest stability were observed for samples prepared with 100% CEC. Previous studies also showed that vermiculites modified with C_16_ and hexadecylpyridinium cations at 100% CEC were stable in the pH range of 4–10, and a lower leaching of organic cations was observed.^45^

#### Characterizations of the Diclofenac-Loaded Samples

**Figure 11:**
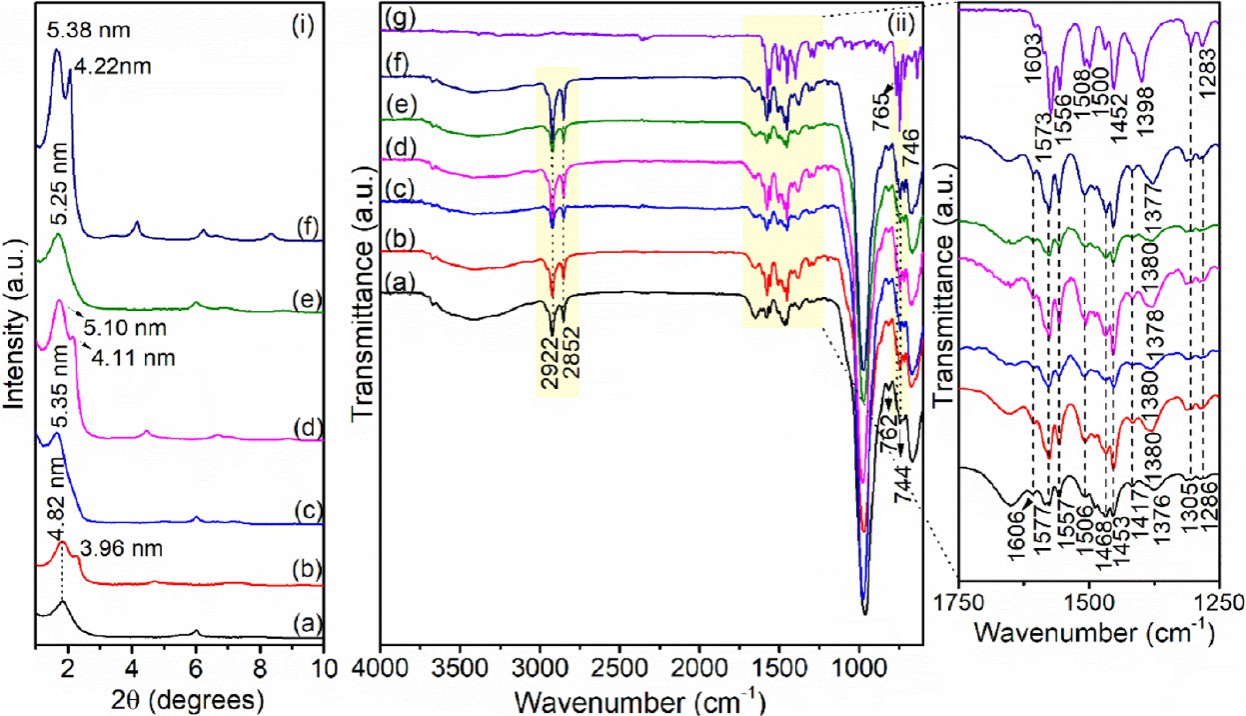
(i) XRD patterns and (ii) FTIR spectra of (a) C_14_-Ver-100%, (b) C_14_-Ver-200%, (c) C_16_-Ver-100%, (d) C_16_-Ver-200%, (e) C_18_-Ver-100%, and (f) C_18_-Ver-200% after diclofenac adsorption (25 °C, pH 6.0 or 8.0 and *C*_i_ = 500 mg L^–1^) and (g) free diclofenac sodium.

XRD and FTIR analysis after adsorption were useful to understand the mechanism of drug/clay minerals interactions. The XRD patterns are shown in Figure 11i. After they interacted with diclofenac, all samples showed an increased basal spacing, suggesting that the drug can be intercalated to access the active adsorption sites. XRD data were also compared with the molecular dimensions of diclofenac, which are 1.0 nm in length, 0.5 nm in width, and 0.4 nm in height,^31^ and are in line with the possible intercalation of the drug or an interlayer rearrangement of surfactant chains after the entrance of diclofenac in both Ver and Hb phases. Similar behavior was observed in the adsorption of naphthalene by organovermiculites.^46^

The FTIR data also provided information about the groups of organophilic vermiculites and diclofenac involved in the adsorption. In the infrared spectra for diclofenac-loaded samples (Figure 11ii), the shift in the ν_as_(CH_2_) band of organophilic samples (initially at 2917 cm^–1^) to higher frequencies (2922 cm^–1^) indicated that the interaction with the drug caused a disorganization or rearrangement of the alkyl chains of the intercalated surfactants,^14,68^ supporting the XRD results. The changes in the CH_2_ stretching frequencies indicate the interaction between long hydrophobic tails of surfactants and the nonpolar moiety of diclofenac.^90,91^ In addition, several bands characteristic of the organic structure of sodium diclofenac were observed (Table S6). Variation in the position of the ν_s_(COO^–^) band, initially at 1398 cm^–1^ for the free drug, to frequencies around 1376–1380 cm^–1^ in loaded diclofenac samples also suggested electrostatic interactions with the carboxylate group during adsorption.^24,26^

#### Mechanism of Interaction

**Figure 12:**
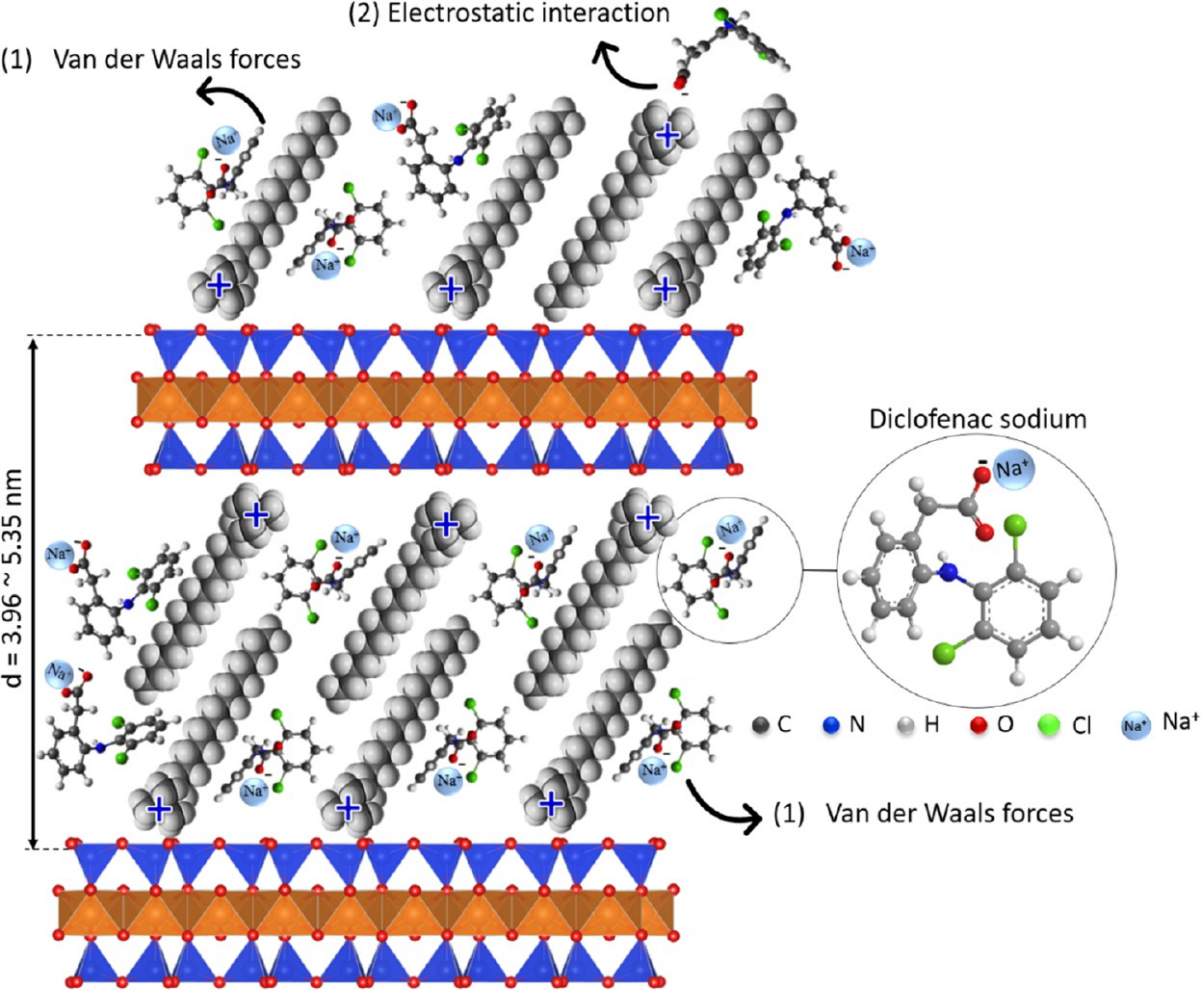
Proposed mechanism for diclofenac adsorption by the organophilic vermiculites through (1) van der Waals forces and (2) electrostatic interactions.

Drawing from the results, a comprehensive schematic of the mechanisms governing diclofenac adsorption on organophilic vermiculites was devised (Figure 12), highlighting the primary involvement of hydrophobic interactions, according to FTIR, Zeta potential results, and study of the effect of pH on adsorption. FTIR data also show that electrostatic interactions can also contribute to the adsorption mechanism. However, considering the negative surface charge of most adsorbents at the adsorption pH, electrostatic interactions should play a less important role in the adsorption mechanism. Furthermore, XRD results showed that drug intercalation occurred in the interlayer space of all organophilic vermiculites. The presence of surfactant on the surface was considered on PCZ results. In this scheme, diclofenac molecules undergo intercalation and interact with (1) alkyl tails of the organic chain through van der Waals interactions and (2) −N^+^–(CH_3_)_3_ surfactant groups through electrostatic interactions. However, mechanism (1) predominates in the adsorption of diclofenac.

#### Reuse Tests

**Figure 13:**
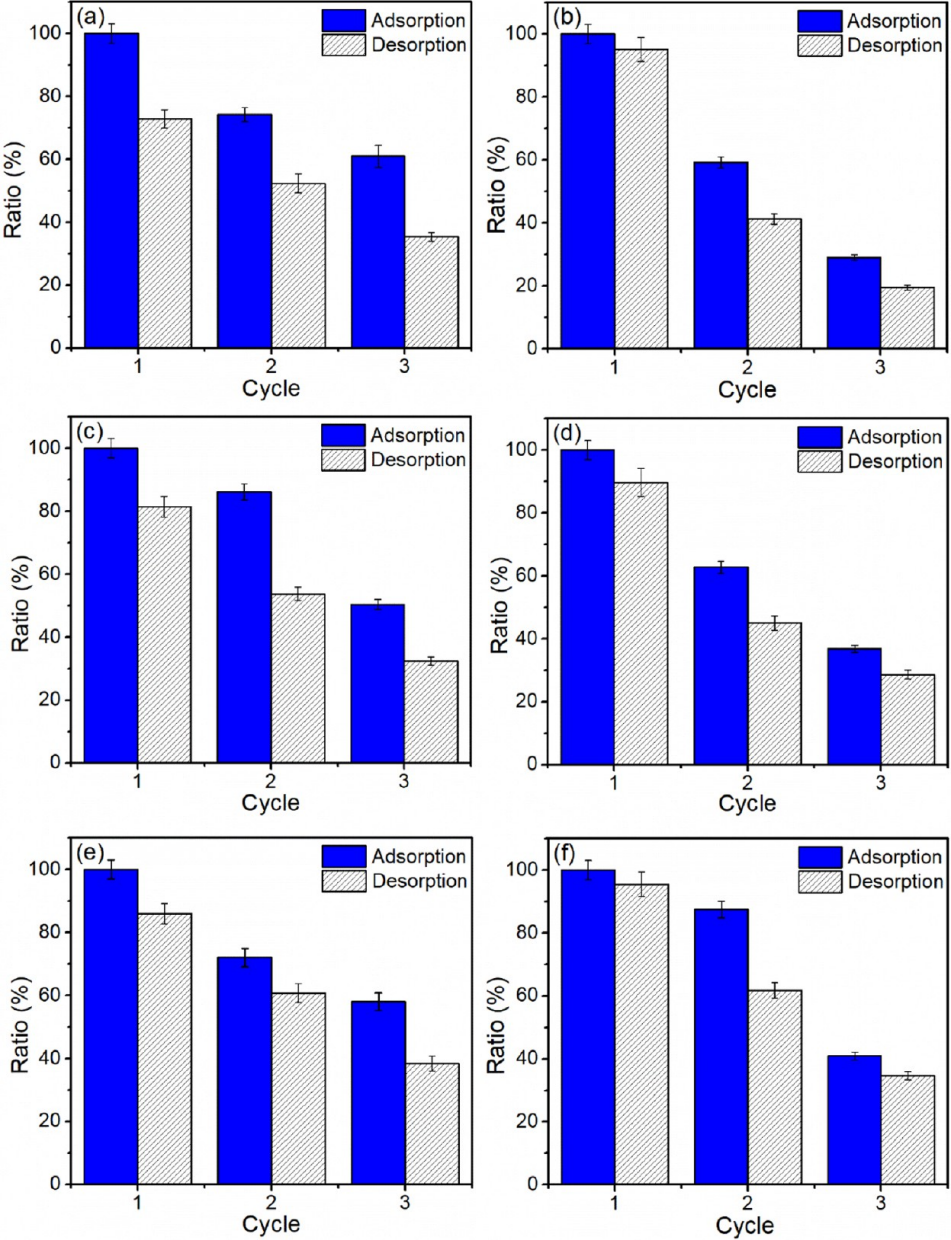
Results of reuse tests performed for (a) C_14_-Ver-100%, (b) C_14_-Ver-200%, (c) C_16_-Ver-100%, (d) C_16_-Ver-200%, (e) C_18_-Ver-100%, and (f) C_18_-Ver-200%.

Several factors influence the selection of an adsorbent, including its production, affinity for the adsorbate, and reusability, among other parameters.^44,92^ Therefore, the regeneration capacity of drug-loaded organophilic vermiculites using ethanol as a desorption agent was evaluated over three adsorption–desorption cycles (Figure 13).

A reduction in adsorption capacity was observed with an increasing number of cycles, which could be attributed to adsorbent losses during the adsorption/desorption and washing processes, as well as the potential reduction or blocking of adsorption sites during regeneration steps.^25,33^ It is worth noting that ethanol molecules may also be adsorbed on organophilic vermiculites during the regeneration process.^33^ In the last cycle, maximum adsorption capacities were maintained at 61.0, 50.4, and 58.0% for C_14_-Ver-100%, C_16_-Ver-100%, and C_18_-Ver-100%, and 29.0, 36.8, and 41.0% for C_14_-200%-Ver, C_16_-Ver-200%, and C_18_-Ver-200%, respectively. The reduction in diclofenac adsorption capacity was higher for organophilic vermiculites prepared with 200% CEC probably due to the lower stability of these samples, in agreement with the stability test results. Additionally, the ethanol used in the washing can also leach surfactants.^93^

## Conclusions

The adsorption of diclofenac on organophilic vermiculites was predominantly influenced by the level of organofunctionalization of the adsorbents. Optimal performance was observed in hybrids prepared with 200% CEC that exhibited a higher organic content compared to those with 100% CEC. However, during reuse tests, these samples did not show enhanced performance. The stability of the samples prepared at 200%CEC was lower compared to that of organosurfactants prepared with 100% CEC. The leaching of surfactants during the adsorption of diclofenac and regeneration with ethanol decreased the performance of the adsorbents in subsequent cycles. Diclofenac was effectively adsorbed into the interlayer region of organophilic vermiculites, and hydrophobic interactions between the tail group of surfactants intercalated into the clay mineral and the nonpolar moiety of the drug played an important role in the adsorption process. However, FT-IR results showed that electrostatic interaction can also occur between the carboxylate group (COO^–^) of diclofenac and the −N^+^–(CH_3_)_3_ surfactants groups.

## Experimental Section

### Materials and Chemicals

A Brazilian vermiculite sample (Ca,Mg-Ver) originating from Santa Luzia (Paraiba, Brazil) was used as the starting material. The chemical composition of Ca,Mg-Ver in mass percentage was previously evaluated by chemical analysis: SiO_2_ (40.08), Al_2_O_3_ (12.35), Fe_2_O_3_ (6.83), TiO_2_ (1.43), CaO (2.32), MgO (18.74), Na_2_O (3.37), K_2_O (2.86) and a mass loss of 11.85% after heating at 950 °C.^94^ Its cation exchange capacity (CEC) was 67 cmol(+) kg^–1^, measured by the ammonium exchange method.^95,96^ All chemicals were used without prior treatment. Sodium chloride (99% purity), ammonium salts tetradecyltrimethylammonium (C_14_Br), hexadecyltrimethylammonium (C_16_Br), and octadecyltrimethylammonium (C_18_Br) bromides (99% purity) were supplied from Sigma-Aldrich. Sodium hydroxide (99% Loba Chemie), nitric acid (75% Vetec), and ethanol (95%, Anidrol) were used. The sodium diclofenac (CAS no. 15307-79-6, MM = 318.13 g mol^–1^) was purchased from Sigma-Aldrich.

### Preparation of Na-Vermiculite (Na-Ver)

The Na-Ver sample was prepared from the Ca,Mg-Ver sample by repeated reactions with a 1.0 mol L^–1^ NaCl solution stirred at 25 °C for 72 h, following a previous procedure^64^ and was carried out in triplicate to ensure complete saturation. The resulting Na-Ver was washed with distilled water until the AgNO_3_ test for chloride anions showed negative results in the supernatant solution and dried at 70 °C for 48 h. Na-Ver was ground and classified by sieving in Tyler sieves (Granutest, Brazil) to obtain a particle size of less than 0.074 mm.

### Preparation of Organovermiculites

Organovermiculites were prepared by reacting between Na-Ver and each alkylammonium salt (C_14_Br, C_16_Br, and C_18_Br) based on a previous procedure.^24,83^ The reaction was carried out as follows: In a Teflon vessel reactor, 4.0 g of Na-Ver was dispersed in 100.0 mL of solution of ammonium salts at 100 and 200% CEC of clay mineral and heated in a microwave reactor (IS-TEC MW reactor model RMW-1, Brazil, with a power of 1100 W 2.45 GHz) for 5 min at 50 °C. Samples were repeatedly washed with distilled water until the AgNO_3_ test for bromide anions showed negative results. The washed organovermiculites were dried at 50 °C at 24 h on a stove under air atmosphere.

### Adsorption Studies

Adsorption tests were performed according to a previous method,^24,48,83^ whose methodology consisted of evaluating the influence of experimental parameters such as pH, adsorbent dosage, contact time, and diclofenac concentration in adsorption.

In a typical procedure, organovermiculite samples were dispersed in 20 mL of diclofenac solution with stirring at 25 °C. The evaluation conditions (pH, adsorbent dosage, drug concentration, and reaction time) were systematically varied according to each test, as listed in Table S7. Adsorption at different pH values was also performed for Na-Ver under the same conditions as used for organophilic vermiculites as a control.

After each test, the adsorbents were separated by centrifugation, and the final drug concentration was determined by UV–vis absorption spectroscopy at 276 nm. The amount of drug adsorbed (*q*) and the drug removal efficiency (*R*%) by organovermiculites was determined by eqs 1 and 2, respectively:
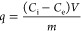
1
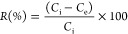
2where *C*_i_ and *C*_e_ are the initial and equilibrium drug concentrations (mg L^–1^), respectively, *m* refers to the mass of the adsorbent (g), and *V* (mL) is the volume of solution.

### Adsorption Models

Adsorption models of Langmuir,^97^ Freundlich,^98^ and Temkin^99^ were applied to adjust and analyze the experimental adsorption data employing the nonlinear method (see Table S8).

The models were also evaluated by standard deviation (SD root-mean-square error),^100^ described in eq 3,
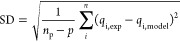
3where *q*_i,exp_, and *q*_i,model_ are the amount of experimental adsorbed drug predicted by the fitted model, *n*_p_ is the number of experiments performed, and *p* is the number of parameters of the fitted model.

### Reuse Studies of Adsorbents

Regeneration of the adsorbents was carried out according to a previous procedure^25^ by dispersing the loaded organophilic diclofenac vermiculites in 50 mL of ethanol stirred for 6 h at 30 °C. After each desorption cycle, the solids were recovered by centrifugation at 7500 rpm, washed with distilled water, and dried at 50 °C to be used for the next adsorption cycle. The readsorption tests were performed under the same conditions as the adsorption experiments.

Drug adsorption/desorption from organovermiculites was calculated as a percentage (%), where the initial adsorption amount was taken as 100%. For instance, the % desorption was calculated by eq 4
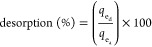
4where *q*_e_a__ and *q*_e_d__ are the quantities of adsorbed and desorbed drugs per unit mass of the adsorbent (mg g^–1^), respectively.

### Evaluation of the Organovermiculites Stability

The stability of organovermiculites was carried out under the same conditions used in the adsorption isotherms, without the presence of diclofenac, to follow changes by the adsorption process, according to the method previously reported.^45^ For this, organovermiculites were suspended in 20 mL of water using the optimal adsorbent dosage. The pH values were adjusted to 6.0 and 8.0 with HCl (0.1 mol L^–1^) and NaOH (0.1 mol L^–1^). Finally, the solids were recovered by centrifugation at 7500 rpm for 10 min and dried at 50 °C for 24 h.

### Characterizations

X-ray diffraction (XRD) data were recorded using an X-ray diffractometer (D8 Advance Bruker-AXS), with the 2θ ranging from 1 to 10° at the scanning, using Cu Kα radiation (λ = 1.5406 nm) at 30 kV and 30 mA. Elemental analysis of C and N was performed using a PerkinElmer PE-2400 microelemental analyzer. Thermogravimetric analyses of organovermiculites were performed using a Discovery TGA instrument under an argon atmosphere with a 100 mL min^–1^ flux from 30 to 800 °C with a heating rate of 10 °C min^–1^. The samples obtained after the stability test were analyzed in TGA Q500 equipment under a N_2_ atmosphere with a 100 mL min^–1^ flux from 30 to 800 °C with a heating rate of 10 °C min^–1^. Fourier transform infrared (FT-IR) spectra were obtained using an IR Prestige-21 spectrometer (Shimadzu) equipped with an ATR accessory, from 4000 to 600 cm^–1^ with a resolution of 4.0 cm^–1^ and 32 scans. The Zeta potentials (ζ) were measured at different pH levels by using a Zetasizer Nano ZS90 (Malvern Instrument). TEM was performed by using a Talos S200 FEI instrument. A voltage acceleration of 200 kV and a current of 4 mA in STEM were used to obtain the HAADF images. SEM was performed by using an FEI Quanta FEG 250 microscope, operating at an accelerating voltage of 15 kV. The nitrogen adsorption isotherms were measured in an ASAP 2420 Micromeritics analyzer. Before measurement, the samples were degassed at 100 °C, and the N_2_ isotherms of adsorption were measured at −196 °C in a *P*/*P*_0_ range of 0.0–1.0. *S*_BET_ value for the C_18_-Ver-100% sample was obtained from the Kr adsorption isotherm in a *P*/*P*_0_ range of 0.0–0.27. The specific surface area (*S*_BET_) of the samples was calculated by Brunauer, Emmet, and Teller (BET) method, while the pore volume and pore diameter were estimated by the Barrett–Joyner–Halenda (BJH) method.

## Supporting Information Available

Basal distance values obtained in TEM; adsorption parameters for equilibrium data calculated according to Langmuir, Freundlich, and Temkin models; comparison of the diclofenac adsorption capacity of organovermiculites with other organoclays; summary of mass losses and temperature intervals for events in the DTG curves and CHN results the samples after stability tests at pH 6 and 8; assignments of the bands in FT-IR spectra of diclofenac-loaded samples; additional experimental details for adsorption evaluation and equations of equilibrium adsorption models; interlayer arrangements of organic chains in the organophilic vermiculites; TEM images for sodium and organophilic vermiculites; N_2_ adsorption–desorption isotherm Na-Ver, C_14_-Ver-100%, and C_16_-Ver-100%; and Kr adsorption isotherm for C_18_-Ver-100% and organovermiculites; graph of zeta potential versus amount of drug adsorbed by the organophilic vermiculites; relation between adsorption capacity and nitrogen content in the adsorbents; and TG/DTG data of the samples after stability tests at pH 6 and 8 (PDF)
