# Supplementary material for: Diclofenac Removal by Alkylammonium Clay Minerals Prepared over Microwave Heating
Source: ACS Omega. 2024 Nov 23;9(49):48256–72. doi: 10.1021/acsomega.4c05763 (PMC11635518; doi:10.1021/acsomega.4c05763)
Supplement: Supplementary file 1 — ao4c05763_si_001.pdf [file ao4c05763_si_001.pdf]

SUPPORTING INFORMATION FOR

Diclofenac removal by alkylammonium clay minerals prepared over microwave heating

Denise B. França[a,1], Alice P. N. Silva[a], Josy A. Osajima[b], Edson C. Silva-Filho[b], Santiago Medina-Carrasco[c], Maria del Mar Orta[d], Maguy Jaber[e], Maria G. Fonseca[a*]

[a]Universidade Federal da Paraíba, Núcleo de Pesquisa e Extensão - Laboratório de Combustíveis e Materiais (NPE - LACOM), Cidade Universitária s/n – Campus I, 58051-900, João Pessoa-PB, Brazil.

[b]Universidade Federal do Piauí, Laboratório Interdisciplinar de Materiais Avançados (LIMAV), Avenida Universitária s/n, 64049-550, Teresina-PI, Brazil.

[c]Universidad de Sevilla, SGI Laboratorio de Rayos X - Centro de Investigación, Tecnología e Innovación de la Universidad de Sevilla (CITIUS), Avenida Reina Mercedes, 4B, 41012, Sevilla, Spain.

[d]Universidad de Sevilla, Departamento de Química Analítica da Facultad de Farmacia, Calle Profesor García González 2, 41012, Sevilla, Spain

[e]Sorbonne Université, CNRS UMR 8220, Laboratoire d'Archéologie Moléculaire et Structurale (LAMS), Case courrier 225, 4 pl. Jussieu, 75252, Paris Cedex 05, France.

*mgardennia@quimica.ufpb.br

[1] *Universidade Federal do Piauí, Laboratório Interdisciplinar de Materiais Avançados (LIMAV), Avenida Universitária s/n, 64049-550, Teresina-PI, Brazil.*

Table S1. Basal distance values obtained from TEM images

Sample	Basal spacing (nm)
C_{14}-Ver-100%	1.1-1.2, 2.6-2.7
C_{14}-Ver-200%	1.1-1.2, 2.6-2.7
C_{16}-Ver-100%	1.1, 2.7-2.8 and 3.9-4.1
C_{16}-Ver-200%	1.0-1.1, 2.9
C_{18}-Ver-100%	1.0-1.2, 2.8-2.9 and 4.2-4.4
C_{18}-Ver-200%	1.0-1.10, 3.3-3.4

Table S2. Adsorption parameters of drug adsorption on organo-vermiculites according to the Langmuir, Freundlich, and Temkin models

Langmuir					
Sample	$q_{e\ (exp)}$ (mg g^{-1})	q_{max} (mg g^{-1})	K_L (10^{-2} L mg^{-1})	R^2	SD (mg g^{-1})
C_{14}-Ver-100%	36.30 ± 1.08	46.70 ± 2.51	1.16 ± 0.16	0.9862	1.62
C_{16}-Ver-100%	52.89 ± 1.05	97.4 ± 7.39	0.30 ± 0.04	0.9913	1.84
C_{18}-Ver-100%	17.90 ± 0.72	21.64 ± 1.00	0.89 ± 0.12	0.9876	0.68
C_{14}-Ver-200%	97.75 ± 1.46	115.10 ± 4.80	1.1 ± 0.13	0.9878	3.63
C_{16}-Ver-200%	110.06 ± 1.65	118.19 ± 4.50	2.39 ± 0.35	0.9836	4.98
C_{18}-Ver-200%	107.97 ± 2.16	115.86 ± 4.50	3.23 ± 0.58	0.9805	5.58

Freundlich				
Sample	n	K_F (mg g^{-1})(mg L^{-1})$^{-1/n}$	R^2	SD (mg g^{-1})
C_{14}-Ver-100%	2.30 ± 0.29	3.06 ± 0.86	0.9383	3.43
C_{16}-Ver-100%	1.61 ± 0.13	1.28 ± 0.36	0.9744	3.15
C_{18}-Ver-100%	2.36 ± 0.17	1.36 ± 0.24	0.9805	0.86
C_{14}-Ver-200%	2.55 ± 0.21	9.21 ± 1.61	0.9750	5.21
C_{16}-Ver-200%	3.39 ± 0.30	19.42 ± 2.72	0.9750	6.15

C_{18}-Ver-200%	3.85 ± 0.47	24.33 ± 4.04	0.9576	8.22
Temkin				
Sample	b_T (10^2 J mol^{-1})	A_T (L mg^{-1})	R^2	SD (mg g^{-1})
C_{14}-Ver-100%	1.34 ± 0.17	0.54 ± 0.24	0.8778	4.84
C_{16}-Ver-100%	1.02 ± 0.21	0.38 ± 0.29	0.7108	10.58
C_{18}-Ver-100%	2.91 ± 0.35	0.54 ± 0.27	0.8813	2.13
C_{14}-Ver-200%	238 ± 46.1	4.51 ± 5.16	0.7423	16.73
C_{16}-Ver-200%	0.84 ± 0.14	33.60 ± 40.8	0.8032	17.26
C_{18}-Ver-200%	0.68 ± 0.06	11.80 ± 6.47	0.9397	9.80

Table S3. Diclofenac adsorption capacity obtained for others organoclays according to the literature

Sample	Surfactant/proportion (% CEC)	Experimental conditions	q_e [c] ($mg\ g^{-1}$)	Reference
Mt[a] (76 cmol(+) kg^{-1})	$C_{16}Br$/400%	pH: 6.5<br>C_i: 10 - 2000 mg L^{-1}<br>Organoclay mass: 100 mg<br>Volume of drug solution: 50 mL<br>t: 24 h<br>T: 25 °C	49.31	Oliveira et al.[1]
	BDTA[c]/400%		60.40	
Bent[b] (75 cmol(+) kg^{-1})	DDP[d]/200%	pH: 6.0<br>C_i: 1-500 mg L^{-1}<br>Organoclay mass: 150 mg<br>V = 20 mL<br>t: 10 min<br>T : 25°C	25.5	França et al.[2]
	HDP[e]/200%	pH: 6.0<br>C_i: 1-500 mg L^{-1}<br>Organoclay mass: 50 mg<br>V = 20 mL	91.13	

		t: 10 min<br>T : 25°C		
Mt<br>(90 cmol(+) kg^{-1})	HDBA[f]/160%	C_i: 20-150 mg L^{-1}<br>Organoclay mass: 50 mg<br>V = 50 mL<br>T : 25 °C	125.55	Chu et al.[3]
Na-Mt<br>(120.0 cmol kg^{-1})	C_{16}Br/200%	pH > 5.0<br>$C_i \approx$ 50 - 1500 mg L^{-1}<br>Organoclay mass = 70 mg<br>V_{sol} (DFNa) = 20 mL<br>t = 24 h	318.13	Sun et al.[4]
Bent<br>(139 cmol(+) kg^{-1})	C_{16}Br/260%	pH: 7.0<br>$C_i \approx$ 159 - 1590 mg L^{-1}<br>Organoclay mass not given<br>V = 40 mL<br>t = 7 days<br>T : 25 °C	388.0	Martinez-Costa et al.[5]
Na-Ver<br>(67 cmol(+) kg^{-1})	C_{14}Br/200%<br>C_{16}Br/200%<br>C_{18}Br/200%	pH 6.0 for C_{16}-Ver-200% and C_{18}-Ver-200%; pH 8.0 for C_{14}-Ver-200%<br>C_i: 1-500 mg L^{-1}<br>Organoclay mass: 50 mg<br>V = 20 mL<br>t: 30 min	97.75<br>110.06<br>107.97	Present study

T : 25°C

[a]Montmorillonite; [b]Bentonite; [c]Benzyldimethyltetradecylammonium; [d]Dodecylpyridinium; [e]Hexadecylpyridinium; [f]Benzyldimethylhexadecylammonium

Table S4. Summary of mass losses and temperature intervals for events in the DTG curves for organovermiculites after stability test

pH	Sample	Event	T (°C)	Mass loss (%)	Total mass loss (%)	Total organic content[a] (%)
6.0	C_{14}-Ver-100%	I	50-115	2.6	19.2	12.8
		II	115-228	3.5		
		III	228-341	7.5		
		IV	341-443	1.2		
		V	443-523	0.6		
		VI	523-762	3.8		
	C_{14}-Ver-200%	I	50-118	2.7	26.2	20.8
		II	118-184	2.8		
		III	184-216	11.3		
		IV	216-345	3.0		
		V	345-518	3.7		
		VI	518-770	2.7		
	C_{16}-Ver-100%	II	50-125	2.2	18.2	13.3
		III	125-221	3.1		
		IV	221-350	9.0		
		V	350-462	1.3		
		VI	522-774	2.6		
	C_{16}-Ver-200%	I	50-118	2.7	29.9	22.8
		II	118-221	7.1		
		III	221-362	13.7		

		IV	362-506	2.0		
		V	506-767	4.4		
	C_{18}-Ver-100%	II	50-133	2.5	20.2	14.9
		III	133-232	3.9		
		IV	232-345	9.4		
		V	345-459	1.4		
		VI	459-528	0.2		
		VII	528-776	2.7		
	C_{18}-Ver-200%	I	50-130	2.3	33.9	27.4
		II	130-266	15.9		
		III	266-380	9.9		
		IV	380-497	1.6		
		V	497-774	4.2		
8.0	C_{14}-Ver-100%	I	50-122	2.7	19.4	12.7
		II	122-221	2.9		
		III	221-340	7.9		
		IV	340-449	1.3		
		V	449-518	0.5		
		VI	518-775	4.1		
	C_{14}-Ver-200%	I	50-117	2.4	24.9	19.0
		II	117-187	2.9		
		III	187-217	2.0		
		IV	217-342	11.2		
		V	342-420	1.9		
		VI	420-513	1.0		

	VII	513-773	3.5		
C_{16}-Ver-100%	I	50-121	2.5	20.3	14.1
	II	121-222	3.3		
	III	222-348	8.9		
	IV	348-446	1.3		
	V	446-519	0.6		
	VI	519-766	3.6		
C_{16}-Ver-200%	I	50-120	2.1	29.4	23.1
	II	120-220	7.7		
	III	220-366	13.6		
	IV	366-506	1.9		
	V	506-770	4.2		
C_{18}-Ver-100%	I	50-120	2.3	21.5	15.7
	II	120-223	4.1		
	III	223-362	10.0		
	IV	362-447	1.1		
	V	447-523	0.5		
	VI	523-769	3.5		
C_{18}-Ver-200%	I	50-118	2.0	33.9	28.1
	II	118-261	16.1		
	III	261-366	10.1		
	IV	366-455	1.5		
	V	455-509	0.5		
	VI	509-766	3.8		

[a]Values obtained from the sum of mass losses, excluding dehydration and dihydroxylation.

Table S5. Results of the CHN elemental analysis of organovermiculites before and after treatment at pH 6.0 and pH 8.0

Sample	Untreated samples				pH 6.0				pH 8.0			
	C (%)	H (%)	N (%)	α[a] (%)	C (%)	H (%)	N (%)	α[a] (%)	C (%)	H (%)	N (%)	α[a] (%)
C_{14}-Ver-100%	11.6	3.2	0.9	15.7	11.5	3.4	0.8	15.8	11.5	3.3	0.8	15.6
C_{16}-Ver-100%	13.0	3.3	0.9	17.2	12.6	3.4	0.7	16.8	12.6	3.4	0.7	16.7
C_{18}-Ver-100%	14.1	3.6	0.8	18.5	13.9	3.6	0.8	18.3	13.9	3.6	0.7	18.2
C_{14}-Ver-200%	18.2	4.3	1.4	23.9	16.5	4.1	1.1	21.7	16.7	4.1	1.1	21.8
C_{16}-Ver-200%	21.3	4.8	1.4	27.5	20.1	4.7	1.2	26.0	20.0	4.4	1.2	25.7
C_{18}-Ver-200%	23.8	5.2	1.4	30.3	23.2	5.1	1.2	29.6	23.3	5.2	1.2	29.7

[a]Total organic content determined from CHN elemental analysis

Table S6. Assignments of the bands in FTIR spectra associated with diclofenac adsorbed in the samples

Wavenumber (cm^{-1})	Assignment[a]	Wavenumber (cm^{-1})	Assignment[a]
1606	ν(ring)	1376~1380	$\nu_s(COO^-)$
1577	$\nu_{as}(COO^-)$	1305	ν_{as}(C-N-C) + CH rock + CH_2 wagging
1557	ν(ring)	1287	CH rock + CH_2 wagging
1506 and 1417	δ(C-N-H) + CH rock (ring)	762	ν(C-Cl) + δ(ring) in-plane
1468 and 1453	ν(C-N) + CH rock (ring)	744	CH wagging (ring)

[a]Iliescu et al.[6] and Lin-Vien et al.[7]

Table S7. Experimental conditions for evaluation of the adsorption of sodium diclofenac by organophilic vermiculites

Parameter evaluated	Experimental conditions			
	pH	m (mg)	t (min)	C_i (mg L^{-1})
pH	6.0; 8.0 and 10.0	25	1440	10
Adsorbent dosage	Fixed[a]	25, 50, 75, 100, 125 and 150	1440	10
Time	Fixed[a]	Fixed[a]	5, 7, 10, 15, 20, 30, 40 and 60	10
Drug concentrations	Fixed[a]	Fixed[a]	Fixed[a]	1, 10, 50, 100, 150, 200, 250, 300, 400, and 500.

[a]Optimal experimental conditions evaluated for each adsorbent.

Table S8. Equilibrium adsorption models

Model	Equation[a]	
Langmuir[8]	$q_e = \frac{q_{max} K_L C_e}{1 + K_L C_e}$	Eq. (S1)
Freundlich[9]	$q_e = K_F C_e^{\frac{1}{n}}$	Eq. (S2)
Temkin[10]	$q_e = \frac{RT}{b_T} \ln(A_T C_e)$	Eq. (S3)

[a]C_e (mg L^{-1}) is the drug equilibrium concentration; q_e (mg g^{-1}) is the equilibrium amount of diclofenac adsorbed on the organoclays; q_{max} (mg g^{-1}) is the maximum adsorption capacity of the adsorbent, assuming the uptake of the monolayer drug by the adsorbent; K_L (L mg^{-1}) is the Langmuir constant; K_F (mg g^{-1}) (mg $L^{-1})^{-1/n}$ and n are Freundlich constants related to the capacity and intensity of adsorption, respectively; b_T is the constant related to the adsorption heat (J mol^{-1}); A_T is the isotherm constant (L mg^{-1}); R is the gas constant (8.314 J mol^{-1} K); T is the absolute temperature (K).

Figure S1. Interlayer arrangements of (a) C_{14}, (b) C_{16}, and (c) C_{18} organic chains in the organophilic samples for (i) vermiculite and (ii) hydrobiotite phases.

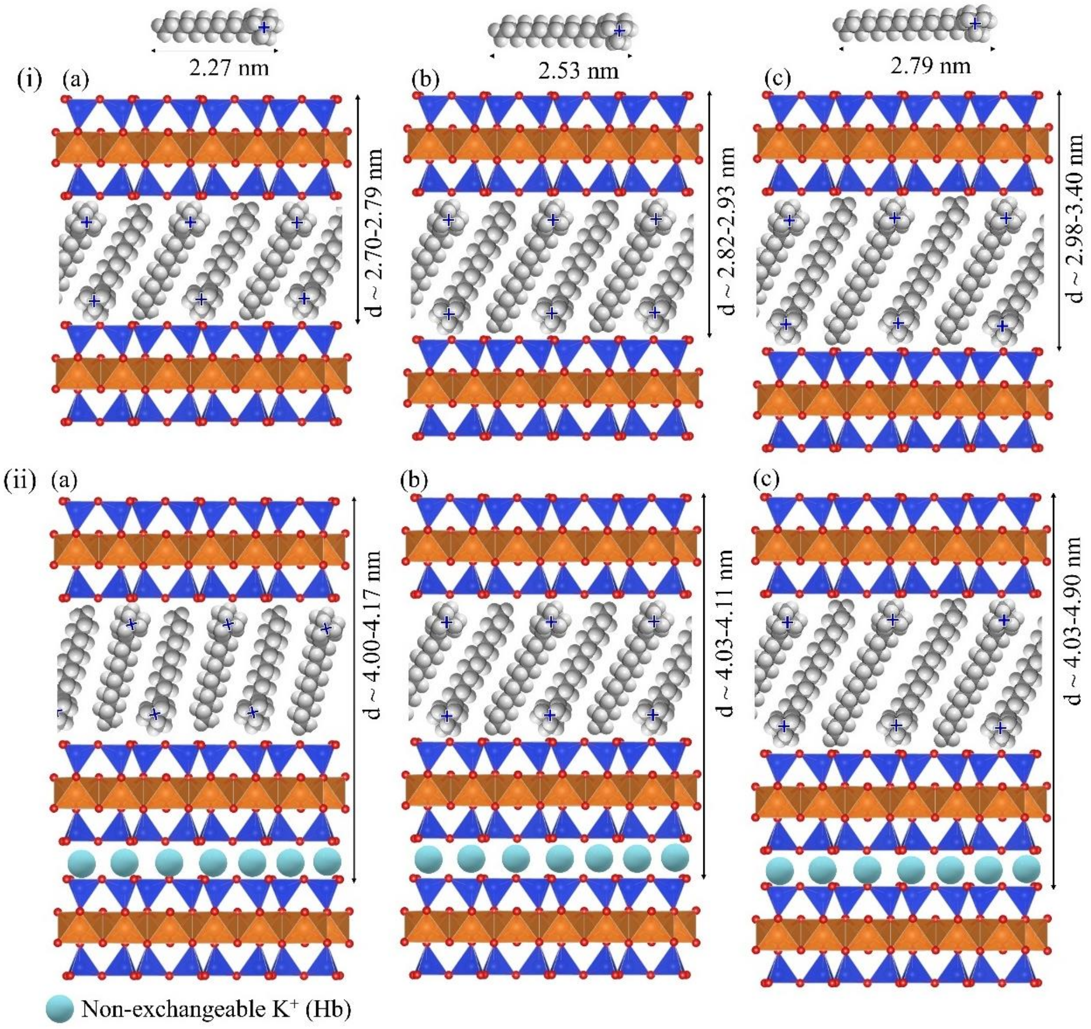

Figure S2. TEM images of (a) Na-Ver, (b) C_{14}-Ver-100%, (c) C_{14}-Ver-200%, (d) C_{16}-Ver-100%, (e) C_{16}-Ver-200%, (f) C_{18}-Ver-100%, (g) C_{18}-Ver-200%.

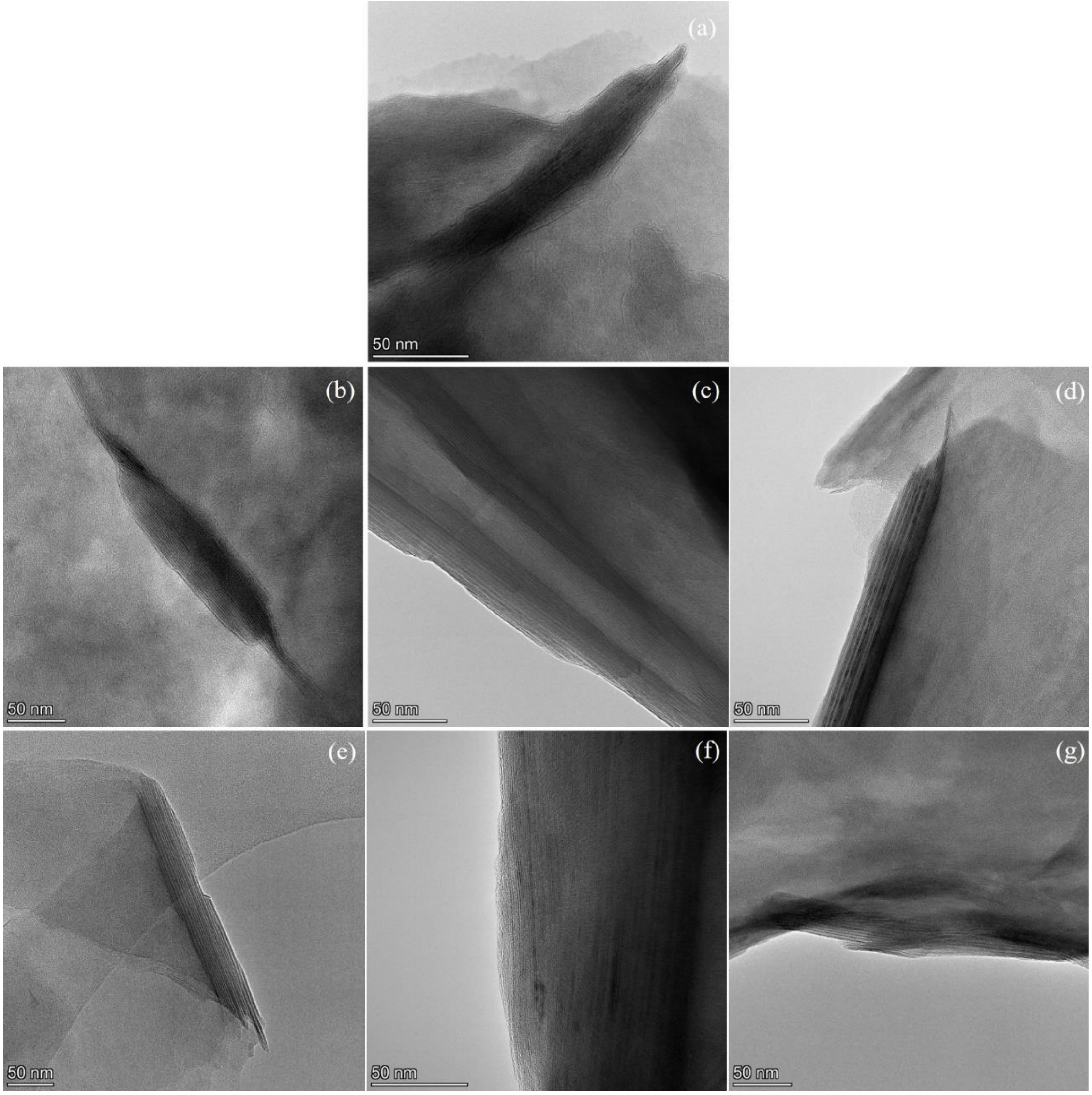

Figure S3. Nitrogen adsorption-desorption isotherms for (a) Na-Ver, (b) C_{14}-Ver-100%, and (c) C_{16}-Ver-100%, and (d) Kr adsorption isotherm for C_{18}-Ver-100%.

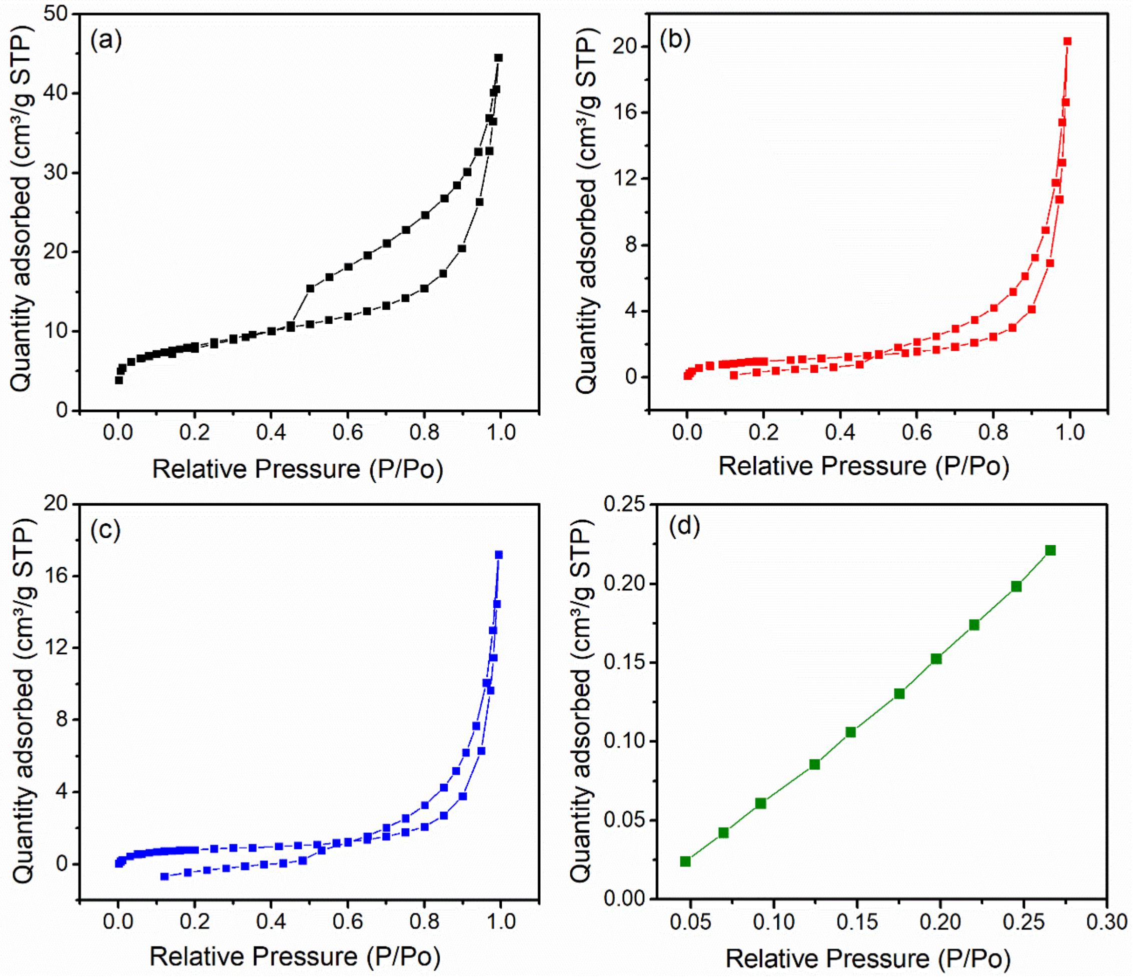

Figure S4. Graph of zeta potential versus amount of drug adsorbed by the organophilic vermiculites (25 °C, 25 mg mass adsorbent, C_i = 10 mg L^{-1}, 24 h, pH 6.0 for C_{16}-Ver-200% and C_{18}-Ver-200%, and pH 8.0 for C_{14}-Ver-100%, C_{14}-Ver-200%, C_{16}-Ver-100%, and C_{18}-Ver-100%.

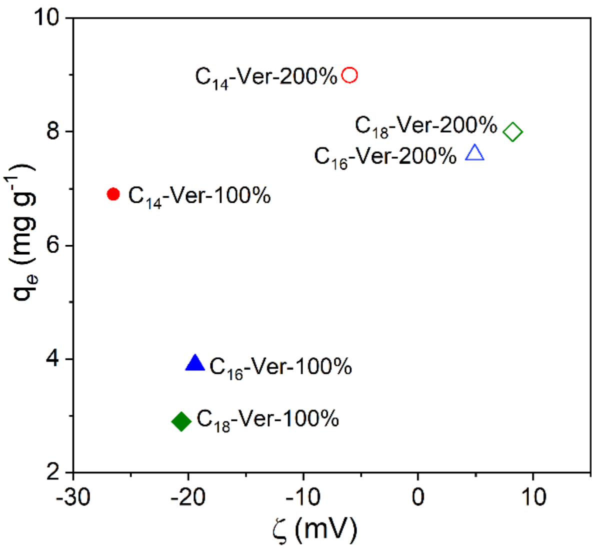

Figure S5. Relation between q_e and nitrogen content in the adsorbents (25 °C, pH 6.0 or 8.0 and C_i = 500 mg L^{-1}).

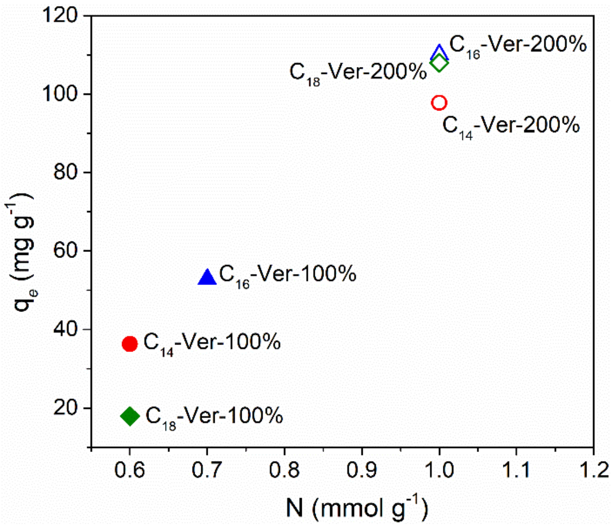

Figure S6. (i) TG and (ii) DTG curves for (a) C_{14}-Ver-100%, (b) C_{16}-Ver-100%, (c) C_{18}-Ver-100%, (d) C_{14}-Ver-200%, (e) C_{16}-Ver-200% and (f) C_{18}-Ver-200% after stability test at pH 6.0, and (iii) TG and (iv) DTG curves for (a) C_{14}-Ver-100%, (b) C_{16}-Ver-100%, (c) C_{18}-Ver-100%, (d) C_{14}-Ver-200%, (e) C_{16}-Ver-200% and (f) C_{18}-Ver-200% after stability test at pH 8.0.

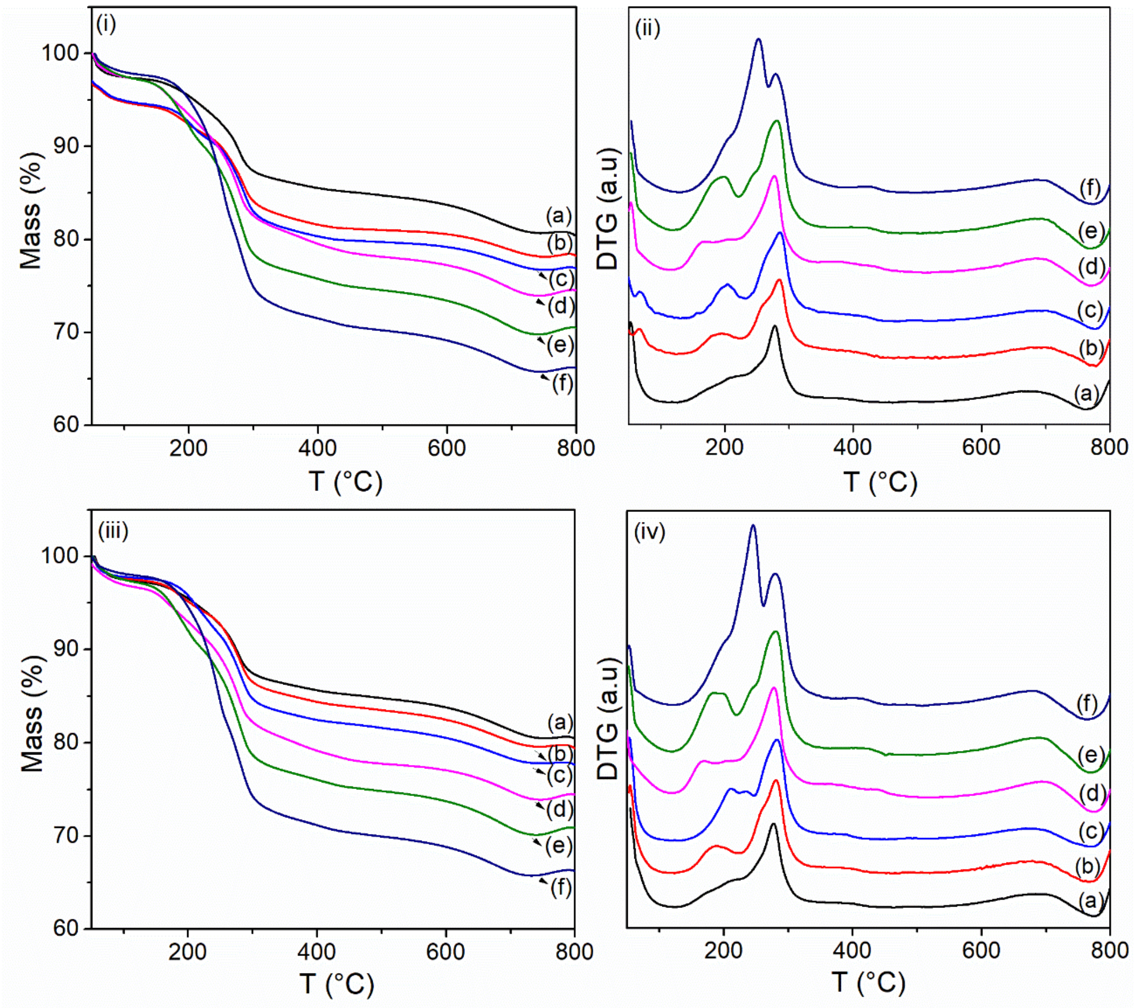

References

(1) Oliveira, T.; Guégan, R.; Thiebault, T.; Milbeau, C. Le; Muller, F.; Teixeira, V.; Giovanela, M.; Boussafir, M. Adsorption of Diclofenac onto Organoclays: Effects of Surfactant and Environmental (pH and Temperature) Conditions. *J. Hazard. Mater.* **2017**, *323*, 558–566, DOI: 10.1016/j.jhazmat.2016.05.001

(2) França, D. B.; Trigueiro, P.; Silva Filho, E. C.; Fonseca, M. G.; Jaber, M. Monitoring Diclofenac Adsorption by Organophilic Alkylpyridinium Bentonites. *Chemosphere* **2020**, *242*, 125109, DOI: 10.1016/j.chemosphere.2019.125109

(3) Chu, Y.; Dai, Y.; Xia, M.; Xing, X.; Wang, F.; Li, Y.; Gao, H. The Enhanced Adsorption of Diclofenac Sodium (DCF) and Ibuprofen (IBU) on Modified Montmorillonite with Benzyldimethylhexadecylammonium Chloride (HDBAC). *Colloids Surf., A* **2024**, *681*, 132764, DOI: 10.1016/J.COLSURFA.2023.132764

(4) Sun, K.; Shi, Y.; Chen, H.; Wang, X.; Li, Z. Extending Surfactant-Modified 2:1 Clay Minerals for the Uptake and Removal of Diclofenac from Water. *J. Hazard. Mater.* **2017**, *323*, 567–574, DOI: 10.1016/j.jhazmat.2016.05.038

(5) Martinez-Costa, J. I.; Leyva-Ramos, R.; Padilla-Ortega, E. Sorption of Diclofenac from Aqueous Solution on an Organobentonite and Adsorption of Cadmium on Organobentonite Saturated with Diclofenac. *Clays Clay Miner.* **2018**, *66*, 515–528, DOI: 10.1346/ccmn.2018.064119

(6) Iliescu, T.; Baia, M.; Kiefer, W. FT-Raman, Surface-Enhanced Raman Spectroscopy and Theoretical Investigations of Diclofenac Sodium. *Chem. Phys.* **2004**, *298*, 167–174, DOI: 10.1016/j.chemphys.2003.11.018

(7) Lin-Vien, D.; Colthup, N. B.; Fateley, W. G.; Grasselli, J. G. *The Handbook of*

Infrared and Raman Characteristic Frequencies of Organic Molecules, 1st ed.; Academic Press: London, 1991.

(8) Langmuir, I. The Adsorption of Gases on Plane Surfaces of Glass Mica and Platinum. *J. Am. Chem. Soc.* **1918**, *40*, 1361–1403, DOI: 10.1021/ja02242a004

(9) Freundlich, H. M. F. Over the Adsorption in Solution. *J. Phys. Chem.* **1906**, *57*, 385–471, DOI: 10.1515/zpch-1907-5723

(10) Temkin, M. J.; Pyzhev, V. Recent Modifications to Langmuir Isotherms. *Acta Physicochim. USSR* **1940**, *12*, 217–222.
